# Impact of the COVID-19 pandemic on access to and delivery of maternal and child healthcare services in low-and middle-income countries: a systematic review of the literature

**DOI:** 10.3389/fpubh.2024.1346268

**Published:** 2024-04-08

**Authors:** Alina Kuandyk (Sabitova), Miguel-Angel Ortega, Magashi Joseph Ntegwa, Antonio Sarria-Santamera

**Affiliations:** ^1^Department of Biomedical Sciences, School of Medicine, Nazarbayev University, Astana, Kazakhstan; ^2^Department of Medicine and Medical Specialties, Faculty of Medicine and Health Sciences, University of Alcalá, Alcalá de Henares, Spain; ^3^Graduate School of Public Policy, Nazarbayev University, Astana, Kazakhstan

**Keywords:** COVID-19, maternal and child healthcare services, low- and middle-income countries, women, paediatric

## Abstract

**Background:**

The COVID-19 pandemic has had a multifaceted impact on maternal and child services and adversely influenced pregnancy outcomes. This systematic review aims to determine the impact of the COVID-19 pandemic on access to and delivery of maternal and child healthcare services in low- and middle-income countries.

**Methods:**

The review was reported following Preferred Reporting Items for Systematic Reviews and Meta-Analyses guidelines. A primary search of electronic databases was performed using a combination of search terms related to the following areas of interest: “impact’ AND ‘COVID-19’ AND ‘maternal and child health services’ AND ‘low- and middle-income countries. A narrative synthesis approach was used to analyse and integrate the results.

**Results:**

Overall, 45 unique studies conducted across 28 low- and middle-income countries met the inclusion criteria for the review. The findings suggest the number of family planning visits, antenatal and postnatal care visits, consultations for sick children, paediatric emergency visits and child immunisation levels decreased compared to the pre-pandemic levels in the majority of included studies. An analytical framework including four main categories was developed based on the concepts that emerged from included studies: the anxiety of not knowing (1), overwhelmed healthcare systems (2), challenges perceived by healthcare professionals (3) and difficulties perceived by service users (4).

**Conclusion:**

The COVID-19 pandemic disrupted family planning services, antenatal and postnatal care coverage, and emergency and routine child services. Generalised conclusions are tentative due to the heterogeneity and inconsistent quality of the included studies. Future research is recommended to define the pandemic’s impact on women and children worldwide and prepare healthcare systems for future resurgences of COVID-19 and potential challenges beyond.

**Systematic review registration:**

PROSPERO (CRD42021285178).

## Introduction

The coronavirus disease (COVID-19) pandemic has had a profound impact on the world, causing not only considerable disruptions to daily life but it has tragically resulted in a significant number of deaths worldwide. According to the World Health Organization (WHO), as of June 5, 2023, there have been more than 767 million confirmed cases of COVID-19, including more than 6.9 million deaths globally (1). Countries around the world have responded to the COVID-19 outbreaks with a range of measures aimed at controlling the spread of the virus and protecting their populations (2). The specific actions taken included imposing lockdowns, movement restrictions, mass testing, contact tracing, mask mandates and hygiene practises (3). Countries have collaborated with each other in sharing data, research and resources and implemented travel restrictions, border closures and mandatory quarantine measures (3).

The COVID-19 restrictions have had a multifaceted impact on healthcare access and delivery. Firstly, routine healthcare services, including non-urgent medical procedures, routine screenings and preventive care, were disrupted due to the re-organisation of the healthcare system to meet the needs of patients diagnosed with COVID-19 (4–6). Secondly, access to healthcare facilities was limited as a result of restrictions on movement and transportation challenges (7, 8). It was also noted that patients tend to avoid seeking healthcare due to fear of contracting COVID-19 in healthcare settings (9). Thirdly, COVID-19 has disproportionately affected healthcare delivery for vulnerable populations and exacerbated existing health disparities (10–12). A WHO survey has recently disclosed that disruptions to healthcare services were predictably greater in low- and middle-income countries (LMICs) than in high-income countries (HICs) (13). Finally, the existing studies have described that outbreaks and responses to them may cause unintentional indirect health ramifications. For instance, the overall use of healthcare services, deliveries in health facilities and malaria admissions decreased by 18% (14), 80% (15) and 40% (15), respectively, during the West African Ebola virus outbreak. It was also estimated that mortality rates from the Ebola virus were comparable to deaths from non-Ebola conditions (16–18). There are concerns that these trends are repeated during the COVID-19 pandemic.

The scale of the COVID-19 pandemic has significantly affected maternal and child services and adversely influenced pregnancy outcomes. A recent systematic review and meta-analysis suggested that maternal mortality, stillbirth, ruptured ectopic pregnancy, and maternal depression increased during the pandemic (19). Other studies report a rise in iatrogenic preterm birth and caesarean delivery amongst infected mothers (20, 21). Furthermore, a number of reports express concerns that the indirect impact of the pandemic might be similar to the direct influence of the virus, specifically in low-income settings (20, 22). A modelling study involving 118 LMICs estimated that the reductions in coverage by maternal and child services might lead to more than a million additional child deaths (23). Another study estimated that a COVID-19-focused approach may have led to 30% additional maternal and child deaths across four different LMICs (24). However, the current understanding of the COVID-19 effects on maternal and child healthcare services is mainly based on pooled estimates of data gathered globally or across HICs, and the number of studies drawing together results from multiple LMICs remains limited (9, 25). Therefore, this systematic review aims to determine the impact of the COVID-19 pandemic on access to and delivery of maternal and child healthcare services in LMICs.

## Methods

The protocol for this review was registered on PROSPERO (CRD42021285178) in advance. This study was reported following the Preferred Reporting Items for Systematic Review and Meta-Analyses (PRISMA) guidelines (26).

### Search strategy

The following five electronic databases were searched: Scopus, Pubmed, Embase, Web of Science, and The Cochrane Central Register of Controlled Trials on October 15, 2021 and updated on June 29, 2023. Search terms combined three overlapping areas with keywords such as ‘impact’ AND ‘COVID-19’ AND ‘maternal and child health services’ AND ‘LMICs’ (see Supplementary Files 1, 2). Publication bias was reduced by searching conference records and unpublished literature using Google Scholar, OpenGrey, EThOS, the British Library Catalogue and Copac theses. In addition, backward and forward citation tracking was adopted to include studies and review records.

### Selection criteria

Studies were eligible if they evaluated the impact of the COVID-19 outbreak on access to and delivery of maternal and child healthcare services in LMICs as defined by World Bank criteria (27). Studies were excluded if they met one of the following conditions: (1) non-research-based articles, such as conference abstracts, commentaries, opinion pieces, book chapters and editorials; (2) are not written using the Latin alphabet, Russian or Kazakh; (3) abstract is not available; (4) or full text is not available.

### Identification and data extraction

Titles and abstracts of identified records were exported to EndNote X8 and screened by AK to exclude irrelevant studies and duplicates. A random sub-sample of 20% of titles and abstracts were screened by a second reviewer (MAO) to ensure the accuracy of selection. Full text articles were inspected again (AK, MAO, MJN and ASS) for relevance according to the inclusion criteria.

Data from included studies were extracted into a spreadsheet by MJN and a random sub-sample of 40% was reviewed by AK and MAO. Discrepancies were addressed by involving a fourth reviewer (ASS). The level of agreement between AK and MAO was 75%, and between AK and ASS was 80%.

### Quality assessment

The methodological quality of the included records was assessed depending on their design. The 14-item Quality Assessment Tool for Observational Cohort and Cross-Sectional Studies (28) was applied in accordance with nine criteria, as five criteria were not applicable. The 12-item Quality Assessment Tool was utilised for Pre-Post (Before-After) Studies With No Control Group (29), the 9-item Quality Assessment Tool was used for Case Series Studies (28), the 7-item Quality Assessment Tool was applied for Mixed-Methods Studies (30) and the 10-item Critical Appraisal Skills Programme (CASP) checklist was adopted for qualitative studies (31) (see Supplementary File 3). AK completed a full quality assessment. MAO ensured the accuracy at this stage by independently assessing 20% of records.

### Data synthesis

A narrative synthesis approach developed by Popay and colleagues (32) was applied to explain and integrate the results.

Firstly, the preliminary synthesis of quantitative data was conducted in order to describe patterns across the included studies grouped by four indicators: impact on maternity service use, impact on maternity service provision, impact on postnatal care and impact on utilisation of child health services. Textual descriptions of studies and tabulation were used as specific tools. A formal meta-analysis was not performed due to considerable heterogeneity in settings and outcome measures.

Secondly, the experiences of service users and healthcare professionals regarding access to and delivery of maternal and child healthcare services during the pandemic were analysed using the Framework Method following the guidelines developed by Gale and colleagues (33). This method includes seven distinct stages: transcription, familiarisation with the data, coding, developing a working analytical framework, applying the analytical framework, charting the data into framework matrix, and interpreting the data. As the review collated data from published studies, the initial stage of transcription was not applicable. The familiarisation stage included reading and rereading the studies included in the review. Further, data from the results sections of included studies were coded and preliminary concepts were defined inductively. Similar concepts were grouped into categories and sub-categories independently by the first author (AK) and were discussed with the other researchers (MAO and ASS) to ensure the range and depth of the coding. The defined categories and sub-categories were then organised into the working analytical framework, which was applied to the results sections of the included studies by systematically coding in a line-by-line manner. Once appropriate codes and categories were assigned, data was charted into the framework matrix by listing illustrative quotations by category and sub-category from each study.

## Results

**Figure 1:**
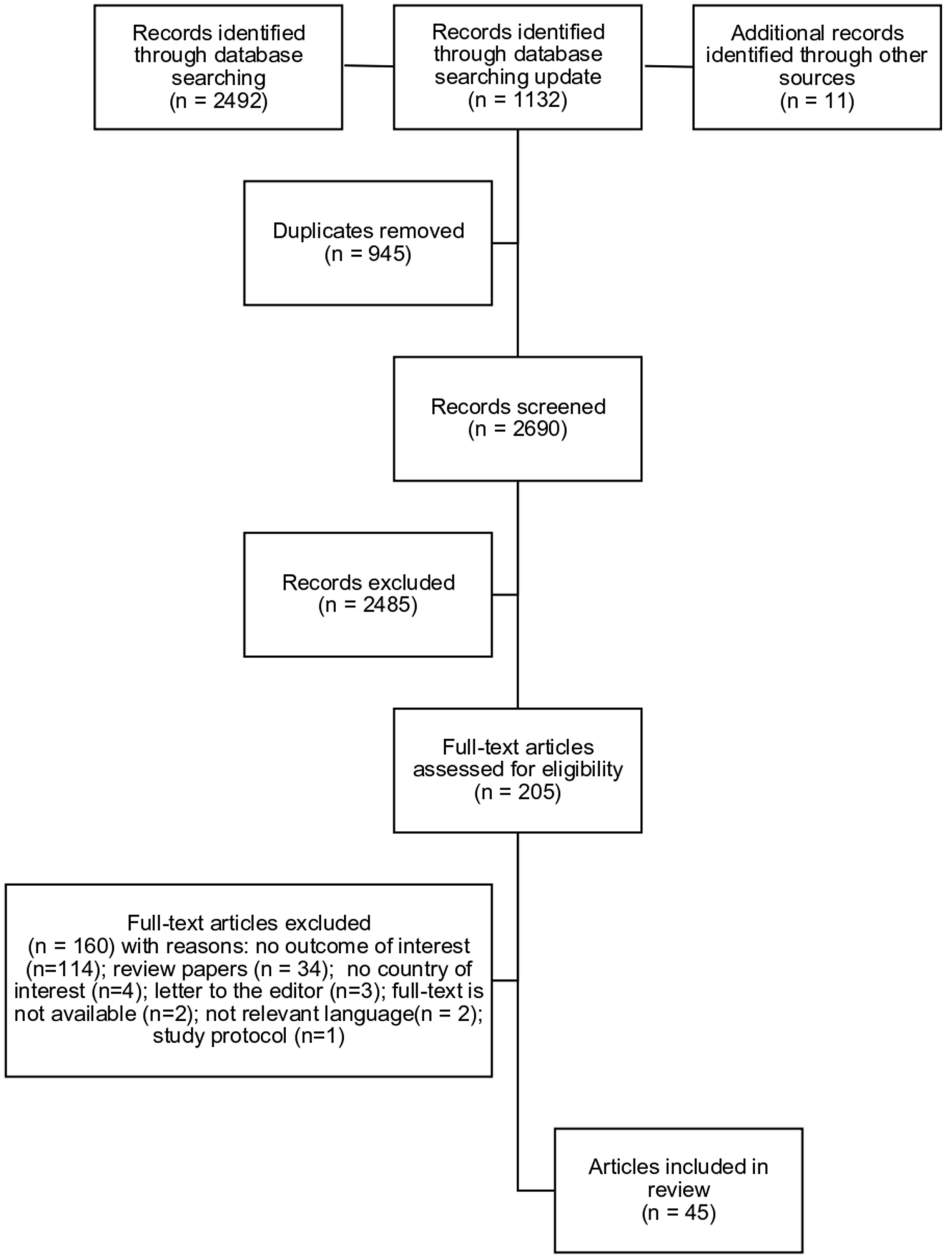
PRISMA flow chart.

The original search yielded 2,492 articles through database searching, 11 through other sources and 1,132 through search update. Overall, 945 articles were removed as duplicates and 2,485 articles were excluded for not meeting the inclusion criteria. The full texts of the remaining 205 papers were examined, 45 of which were included to the review. The detailed selection process is presented in the PRISMA flow diagram below (Figure 1).

### Overview of included studies

Studies were published between 2020 and 2023 solely in English. Overall, 14 studies reported data from four low-income countries (34–47), 21 studies were focused on 13 lower-middle income countries (48–68), seven studies were conducted in five upper-middle income countries (69–75) and three were multi-centred (76–78). Out of 45 included studies, 11 studies were cross-sectional (41, 45, 48, 51, 55–57, 65, 69, 70, 76), 14 were pre-post studies (34, 38, 49, 50, 52, 53, 56, 62, 64, 72–75, 77), nine were time-series (35, 37, 39, 43, 58, 59, 67, 71, 78), five were mixed methods (36, 42, 47, 61, 63) and six were qualitative (40, 44, 46, 54, 60, 68). The included studies’ characteristics are summarised in Table 1.

**Table 1:** Characteristics of included studies.

N	Authors, year	Country (income group)	Aim	Study design	Study population	Sampling	Sample size	Outcome(s) reported	Findings
1	Abdela et al., 2020 (34)	Ethiopia (low-income)	To assess the effect of prevention measures on essential healthcare services at Dessie Referral Hospital	Pre-post	Patients attending different essential healthcare services	Census	Not reported	Number of mothers delivering at the hospital	No difference
								Family planning visits	Decreased
								Antenatal care visits	Decreased
								Neonatal admissions	Decreased
								Childhood emergency visits	Decreased
2	Abdul-Mumin et al., 2021 (48)	Ghana (lower middle-income)	To describe the impact of the COVID-19 pandemic on new born care by comparing morbidity and mortality between the COVID-19 era and the preceding year in the Neonatal Intensive Care Unit (NICU) at Tamale Teaching Hospital, Ghana	Cross-sectional	Neonates admitted to the Neonatal Intensive Care Unit (NICU)	Census	2,901	Admissions of inborn neonates	Decreased
								Neonates born at home	Decreased
								Proportion of referrals to the NICU from other facilities	Increased
								Admissions due to neonatal infections	Decreased
								Admissions due to prematurity and complications, and neonatal jaundice	Increased
3	Abebe et al., 2021 (35)	Ethiopia (low-income)	To assess the impact of COVID-19 on the trends of nonCOVID follow-up visits and admissions at Tikur Anbessa Specialised Hospital (TASH), Addis Ababa, Ethiopia	Time-series	Patients at TASH	Census	12,314 (follow-up visits) and 5,693 (hospital admissions) – (General data)	Paediatric emergency admissions	Decreased
								Admissions from the general paediatric follow-up clinics	Decreased
4	Ahmed et al., 2021 (77)	multi-centred Bangladesh (lower middle-income) Nigeria (lower middle-income) South Africa (upper middle-income)	To assess the disruption in utilisation of maternal, neonatal and child health care as a result of the COVID-19 pandemic in three LMICs	Pre-post	Users of the maternal, neonatal and child health services	Census	Not reported	Attendance of antenatal care	Decreased
								Attendance of family planning clinics	Decreased
								Child immunisation	Decreased
								Facility vaginal delivery rates	Decreased in Bangladesh Mixed in Nigeria and South Arica
								Caesarean section delivery rates	Decreased in Bangladesh Mixed in Nigeria and South Arica
5	Akuaake et al., 2020 (69)	South Africa (upper middle-income)	To describe and compare the effect of the level 5 national COVID-19 lock-down measures on the workload and case mix of paediatric patients presenting to a district-level emergency centre in Cape Town, South Africa	Cross-sectional	Patients less than 13 years of age that presented to the emergency centre of Mitchells Plain Hospital	Convenience	9,982	Children emergency centre visits	Decreased
								Presentations of respiratory diseases, infectious diseases and injuries	Decreased
6	Assefa et al., 2021 (76)	multi-centred Burkina Faso (low-income) Ethiopia (low-income) Nigeria (lower middle-income)	To characterise the impacts of the COVID-19 pandemic on the interruptions on health services from the perspectives of both HCPs and community members in three sub-Saharan African countries, Burkina Faso, Ethiopia, and Nigeria	Cross-sectional	Healthcare providers and community members	Not reported	900 healthcare providers and 1797 community members	Interruptions in antenatal care	Increased
								Interruptions in folate supplementation	Increased
								Interruptions in family planning	Increased
								Interruptions in maternal and child services	Increased
7	Baloch et al., 2021 (49)	Pakistan (lower middle-income)	To assess the utilisation of reproductive, maternal, neonatal, and child health services at the primary healthcare level during the first wave of the COVID-19 outbreak in Sindh, Pakistan	Pre-post	Users of the reproductive, maternal, neonatal, and child health services	Convenience	Not reported	First antenatal visits	Decreased
								Number of pregnant women receiving the second dose of tetanus toxoid vaccine during pregnancy	Decreased
								Number of normal vaginal deliveries	Decreased
								Family planning visits	Decreased
								Number of children receiving their scheduled vaccination	Decreased
8	Singh et al., 2021 (50)	India (lower middle-income)	To quantify the potential impact of the COVID-19 pandemic on maternal and child health services in the state of Uttar Pradesh, India	Pre-post	Users of the maternal and child public health facilities of District Sant Kabir Nagar in Uttar Pradesh, India.	Not reported	Not reported	Number of institutional deliveries	Decreased
								Attendance of antenatal care services	Decreased
								Immunisation services	Decreased
9	Shapira et al., 2021 (78)	multi-centred Cameroon (lower middle-income) Democratic Republic of Congo (low-income) Liberia (low-income) Malawi (low-income) Mali (low-income) Nigeria (lower middle-income) Sierra Leone (low-income) Somalia (low-income)	To quantify the disruption of maternal and child health services during the COVID-19 pandemic using nation- ally comprehensive administrative data in eight sub-Saharan African nations	Time-series	Users of the maternal and child health services	Census	9,499,075	Number of outpatient department consultations	Decreased
								Number of child vaccinations	Decreased
								Number of institutional deliveries	Decreased (in 5 countries)
								Attendance of antenatal care services	Decreased
								Postnatal care visits	Decreased
	Shakespeare et al., 2021 (51)	Zimbabwe (lower middle-income)	To compare maternal and perinatal outcomes before and after lockdown was implemented	Cross-sectional	Users of the government tertiary level maternity unit in Bulawayo, Zimbabwe	Not reported	Not reported	Workload	No difference
								Number of deliveries	Decreased (not significant)
								Number of Caesarean section deliveries	Decreased (not significant)
								Attendance of antenatal care services	Decreased
								Maternal mortality	No difference
								Stillbirth rate	Decreased (not significant)
								Number of early neonatal deaths	Increased (not significant)
11	Rahul et al., 2020 (51)	India (lower middle-income)	To analyse the impact of this pandemic on the management of paediatric surgical cases at four tertiary care centres in Northern India.	Pre-post	Paediatric patients who underwent surgery	Census	100	Total emergency cases	Decreased
								Number of patients who left against medical advice	Increased
12	Qureshi et al., 2021 (53)	India (lower middle-income)	To evaluate the extent by which the lockdown, imposed by the government, has impacted the activity of admissions to the tertiary maternity hospital in Srinagar	Pre-post	Patients admitted to the tertiary maternity hospital in Srinagar	Census	Not reported	Total number of emergency admissions	Decreased (significant)
								Number of patients admitted with intrauterine device	Increased (significant)
								Number of patients with eclampsia	Increased (significant)
								Number of patients admitted with ectopic abruptions, obstructed labour and postpartum haemorrhage	No difference
13	Muhaidat et al., 2020 (70)	Jordan (upper middle-income)	To identify how the lockdown circumstances in Jordan have affected antenatal care provision to pregnant women across the country	Cross-sectional	Women residing in Jordan who are currently pregnant	Not reported	944	Attendance of antenatal care	Decreased (significant)
14	Pires et al., 2021 (36)	Mozambique (low-income)	To assess the impact of Covid-19 pandemic Government restrictions on access to maternal and child healthcare services	Mixed-methods	Users of maternal and child health care unit for survey and 19 females participants for interviews (mothers, pregnant women, traditional birth attendants and nurses)	Not reported	Qualitative component: 19 (10 users and 9 nurses)	Number of home deliveries	Increased (not significant)
								Number of pregnant women attending their first antenatal visit	Increased (not significant)
								Number of women completing four antenatal visits	Increased (not significant)
								Number of well-baby visits	Increased (not significant)
								Number of elective Caesarean sections	Decreased (not significant)
								Number of hospital deliveries	Decreased (significant)
15	Onchonga et al., 2021 (54)	Kenya (lower middle-income)	To understand the health-seeking behaviour of women who were pregnant during the onset of the COVID-19 pandemic in Kenya	Qualitative	Women who had attended at least one antenatal care clinic in a county referral hospital in Kenya	Purposive	26	Attendance of antenatal care	Decreased
								Delays in reaching the health facility	Increased
								Delays related to the experience of pregnant women at healthcare facilities	Increased
16	Ogundele et al., 2020 (55)	Nigeria (lower middle-income)	To assess early effects of the COVID-19 pandemic on paediatric surgical practise in Nigeria	Cross-sectional	Paediatric surgeons (consultants and senior registrars) currently practising in Nigeria	Not reported	74	Number of elective surgeries	Decreased
								Number of emergency surgeries	Decreased
17	Doubova et al., 2021 (71)	Mexico (upper middle-income)	To estimate the overall effect of the pandemic on essential health service use and outcomes in Mexico, describe observed and predicted trends in services over 24 months, and to estimate the number of visits lost through December 2020	Time-series	Users of the Mexican Institute of Social Security	Not reported		Number of antenatal care visits	Decreased
								Number of facility deliveries	Decreased
								Caesarean section rate	No difference
								Number of consultations for sick children	Decreased
								Number of childhood vaccinations	Decreased
18	Burt et al., 2021 (37)	Uganda (low-income)	To quantify the indirect impact of COVID-19 on maternal, neonatal and childhood outcomes at KNRH in Kampala	Time-series	Users of the Kawempe National Referral Hospital	Not reported	14,401 antenatal care attendances, 33,499 deliveries, 111,658 attendances for childhood services and 57,174 sexual and reproductive health service attendances	Number of antenatal care visits	Decreased
								Number of attendances for prevention of mother-to-child transmission of HIV	Decreased
								Number of women treated for high blood pressure, eclampsia and pre-eclampsia, adverse pregnancy outcomes (stillbirths, low-birth-weight and premature infant births)	Increased
								Rate of neonatal unit admissions	Increased
								Rate of neonatal deaths	Increased
								Maternal mortality	No difference
								Immunisation clinic attendance	Decreased
19	Caniglia et al., 2021 (72)	Botswana (upper middle-income)	To evaluate the association between the COVID-19 lockdown and the risk of adverse birth outcomes in Botswana	Pre-post	Women who delivered a singleton baby after at least 24 weeks’ gestation in 2017–2020 between January 1 and July 20	Census	68,448	Number of births	No difference
								Number of antenatal visits	No difference
								Risk of any adverse birth outcome	Decreased
								Risk of any severe birth outcomes	Decreased
20	Desta et al., 2021 (38)	Ethiopia (low-income)	To assess the impacts of COVID-19 on essential health services delivery in Tigray, Northern Ethiopia	Pre-post	Users of essential health services in Tigray	Purposive	Not reported	Family planning acceptance rate	Decreased
								Number of antenatal care visits	Decreased
								Number of women who received comprehensive abortion care	Decreased
								Number of children under 2 years of age who have received second dose of measles	Decreased
								Number of institutional deliveries	Increased
								Number of caesarean section deliveries	Increased
								Number of still births	Increased
								Number of children who received all vaccine doses before 1st birthday	Increased
								Number of under 5 children screened and had moderate and severe malnutrition	Increased
21	Hategeka et al., 2021 (39)	Democratic Republic of the Congo (low-income)	To evaluate the impact of the pandemic on the use of essential health services during the first wave of the pandemic in Kinshasa	Time-series	Users of health facilities across Kinshasa	Not reported	3,467,713	The use of maternal health services	No difference
								Child immunisation	No difference
22	Pillay et al., 2021 (73)	South Africa (upper middle-income)	To assess the impact of COVID-19 and restrictions imposed to limit viral transmission on routine health services in South Africa	Pre-post	Users of health services in South Africa	Not reported	Not reported	Access to contraceptives	Decreased
								Number of first antenatal care visits	No difference
								Number of deliveries in public health facilities	Increased
								Maternal mortality	Increased
								Neonatal deaths	Increased
								Child immunisation	Decreased
23	Hailemariam et al., 2021 (40)	Ethiopia (low-income)	To explore COVID-19 related factors influencing antenatal care service uptake in rural Ethiopia	Qualitative	Pregnant women residing in rural districts of Bench-Sheko Zone, and healthcare providers working in the local health care facilities	Purposive	44 pregnant women and 9 healthcare providers	Health facility barriers	Increased
								Quality of care	Decreased
								Difficulties in accessing maternal health care	Increased
								Anxiety	Increased
								Fear of getting COVID-19	Increased
24	Goyal et al., 2021 (56)	India (lower middle-income)	To assess the indirect effect of the COVID-19 pandemic on the health of pregnant women and foetal-maternal outcomes	Pre-post	Users of the e Department of Obstetrics and Gynaecology at All India Institute of Medical Sciences	Not reported	Not reported	Number of admissions	Decreased
								Number of institutional deliveries	Decreased
								Number of high risk pregnancies	Increased
								Number of antenatal care visits	Decreased
25	Enyama et al., 2020 (57)	Cameroon (lower middle-income)	To describe the impact of the COVID-19 pandemic on the clinical activity of paediatricians	Cross-sectional	Paediatricians practising in Cameroon	Not reported	101	Number of paediatric outpatient consultations	Decreased
								Use of telehealth	Increased
26	Enbiale et al., 2021 (41)	Ethiopia (low-income)	To study the effect of preventive COVID-19 measures on essential healthcare services in selected primary and tertiary care settings of Amhara region, Ethiopia	Cross-sectional	Users of healthcare facilities at Amhara region	Not reported	Not reported	Number of family planning visits	Decreased (not significant)
								Number of institutional deliveries	Increased
								Child immunisation	No difference
								Number of institutional deliveries	Decreased
27	Yadollahi et al., 2022 (58)	Iran (lower middle-income)	To assess the impact of the COVID-19 pandemic on maternal healthcare indices and care providers’ performance	Time-series	Users of the Shiraz University of Medical Sciences, Shiraz, Southern Iran		63,000 pregnant women	Number of preconception healthcare visits	Decreased
								Number of first routine laboratory tests	Decreased
								Number of prenatal care visits	Decreased
								Number of first and second trimester sonography	Decreased
28	Tilahun et al., 2022 (42)	Ethiopia (low-income)	To examine the effects of the pandemic (COVID-19) on maternal and child health service utilization	Mixed-methods	Qualitative component: decision-makers, health workers, patients and delegates from non-governmental organisations	Qualitative component: purposive Quantitative component: not reported	Qualitative component: 74	Accessibility and quality of routine health services	Decreased
								Utilisation of maternal and child health services	Decreased
								Number of challenges on the commitment of health worker	Increased
								Resources supply	Decreased
								Contraceptive acceptance rate	Increased(not significant)
								Antenatal care coverage	Decreased (not significant)
								Number of skilled deliveries	No difference
								Postnatal care coverage	Increased (significant)
								Child immunisation	Increased (not significant)
29	Tikouk et al., 2023 (59)	Morocco (lower middle-income)	To evaluate the impact of the COVID-19 pandemic on antenatal indicators in the region of Guelmim Oued Noun, Morocco	Time-series	Users of public health services at the region of Guelmim Oued Noun, Morocco	Not reported	Not reported	Antenatal recruitment rate	Decreased
								Recruitment rate of pregnant women visits in the 1st quarter of pregnancy	Decreased
								Prenatal visit completion rate	Decreased
								Average number of visits per pregnancy	Decreased
30	Thahir et al., 2023 (60)	Indonesia (lower middle-income)	To explore the experiences of Indonesian mothers and midwives from a rural regency regarding maternal and child health services delivery during the pandemic	Qualitative	Mothers and midwifes in four sub-districts in Banggai, Indonesia	Random	21 mothers and 6 midwives	Health service change	Service relocation, reduces services, health service changes specific to COVID-19, support within the health service for mothers affected by the pandemic
								Perceived barriers to service delivery	Mothers’ perceived barriers for accessing service, midwives’ perceived barriers for providing service
								Family impact	Financial impact, emotional impact
31	Sinha et al., 2022 (61)	India (lower middle-income)	To estimate utilisation of maternal, perinatal healthcare services after the lockdown was implemented in response to the COVID-19 pandemic compared to the period before.	Mixed-methods	Women who delivered before and after lockdown	Not reported	Quantitative component: 199 Qualitative component: 25	Number of antenatal care visits	Decreased
								Proportion of institutional deliveries	Decreased
								Faces issues	Fear of contracting COVID-19, poor quality of services, lack of transportation, financial constraints, poor mental conditions (feeling down, depressed or hopeless)
32	Sharma et al., 2023 (62)	India (lower middle-income)	To document the impact of COVID-19 on essential maternal and child health services in India based on the national Health Management Information System	Pre-post	Users of maternal and child health services	Census	Not reported	Antenatal care registrations	Decreased
								Number of pregnant women provided with emergency obstetric care	Decreased
								Number of institutional deliveries	Decreased
								Number of home deliveries	Increased
								Child immunisation	Increased
33	Requena-Mullor et al., 2022 (74)	Dominican Republic (upper middle-income)	To analyse the differences in perinatal outcomes and birth characteristics in two groups of pregnant women, and whether these differences are due to changes in pregnancy monitoring because of the COVID-19 situation	Pre-post	Women who gave birth before and during the pandemic	Census	Overall: 1109 Before pandemic: 496 During pandemic: 613	Number of antenatal visits	Decreased
								Number of instrumental and caesarean deliveries	Increased
								Skin-to-skin contact after birth	Decreased
								Introduction of early breastfeeding	Decreased
34	Padhye et al., 2022 (63)	India (lower middle-income)	To present users’ and providers’ perspectives about the effect of the pandemic on maternal health services in select districts of Assam	Mixed-methods	Service users and providers	Quantitative component: random Qualitative component: purposive	Quantitative component: 114 pregnant and recently delivered mothers Qualitative component: 38 healthcare providers and 18 Village Health Sanitation and Nutrition Committee members and	Access to antenatal care	Not changed
								Transportation issues	Increased
								Expenses for healthcare services	Increased
								Opportunities to participate in health planning at the local level	Decreased
								Proportion of caesarean section deliveries	Increased
								Number of still-births	Increased
35	Millimouno et al., 2023 (64)	Guinea (lower middle-income)	To analyse the effect of COVID-19 on routine maternal and neonatal health services in Guinea	Pre-post	Users of maternal and neonatal health services in three referral hospitals - Hôpital National Ignace Deen (HNID), Hôpital Regional de Mamou (HRM) in Mamou and Institut de Nutrition et de Santé de l’Enfant (INSE)	Exhaustive	Not reported	Mean monthly number of deliveries	Decreased in HNID Increased in HRM
								Obstetric complications	Increased in HNID Decreased in HRM
								Mean monthly number of maternal deaths	Increased in HNID and HRM
								Mean monthly number of neonatal admissions	Decreased in INSE
								Mean monthly number of neonatal deaths	Decreased in INSE
36	Mhajabin et al., 2022 (65)	Bangladesh (lower middle-income)	To present the effect of the early phase of COVID-19 on the coverage of essential maternal and newborn health services in a rural subdistrict of Bangladesh	Cross-sectional	Group 1: women who were on the third trimester of pregnancy during April–June 2020 Group 2: women who were on the third trimester of pregnancy during August–October 2019 Group 3: women who gave birth during April–June 2020 Group 4: women who gave birth in August–October 2019	Random	Group 1: 111 Group 2: 115 Group 3: 163 Group 4: 166	Number of women received at least one antenatal care service from a medically trained provider	Decreased (not significant)
								Number of visits by a medically trained provider	Increased (not significant)
								Birth, antenatal care, postnatal care and essential newborn care coverage	No difference
37	Lydon et el., 2022 (36)	Mozambique (low-income)	To measure the effects of the COVID-19 on maternal and perinatal health services and outcomes in Mozambique	Time-series	Users of public health facilities providing antenatal or maternity services in Nampula Province	Census	Not reported	Number of first antenatal care visits	Increased
								Fourth antenatal care visits completed	No difference
								Number of facility deliveries	Increased
								Adverse birth outcomes	No difference
38	Kabagenyi et al., 2022 (43)	Uganda (low-income)	To understand the extent to which COVID-19 interrupted access and utilisation of FP information and services during the lockdown in Uganda	Qualitative	Policy makers, implementers, researchers and family planning service providers	Purposive	21	Disrupted service delivery	No outreaches conducted, limited availability of family planning commodities, low family planning access and utilisation and inadequate human recourses or health workers
								Mobility hindrances	Difficulty in finding transport means, high cost of transport and restricted movement
								Responsive reproductive health services	Referral services offered to family planning clients and distribution of family planning commodities
								Financial related disruptions	Loss of employment and unemployment
39	Goyal et al., 2022 (66)	India (lower middle-income)	To assess the difficulties faced by the pregnant women in seeking appropriate antenatal care due to the restrictions imposed during the COVID-19 pandemic	Cross-sectional	Pregnant women enlisted in the study area just before the enforcement of the lockdown	Multistage (convenience, purposive and census)	1,374	Perceived difficulties	Due to the restrictions in getting adequate nutrition (76.5%), accessing transportation facilities (35.4%), consultations from doctors (22.4%), getting an ultrasonography scan (48.7%). Overall, 21.9% of women could not access safe abortion services. Only 3.6% of respondents ever took any teleconsultation services offered by the government. Most of them felt unsatisfied compared with routine visits (77.5%).
40	Gebreegziabher et al., 2022 (45)	Ethiopia (low-income)	To assess trends in selected maternal and child health services performance in the context of COVID-19 pandemic	Cross-sectional	Users of maternal and child health services in Addis Ababa City	Not reported	Not reported	Number of postnatal care visits	Decreased
								Number of new contraceptives accepters	Decreased
								Safe abortion care	Decreased
								Number of under-5 years old children treated for pneumonia	Decreased
41	Emmanuel et al., 2022 (67)	Pakistan (lower middle-income)	To appraise the effects of containment and lockdown policies on reproductive, maternal, newborn and child health service utilisation in Pakistan	Time-series	Users of all public reproductive, maternal, newborn and child health services	Census	Not reported	Family planning visits	Decreased
								Number of antenatal care visits	Decreased
								Number of institutional deliveries	Decreased
								Number of caesarean sections	Decreased
								Number of postnatal care visits	Decreased
								Child immunisation	Decreased
42	Bliznashka et al., 2022 (46)	Mozambique (low-income)	To understand caregiver utilisation and provider delivery of child health services since the start of the pandemic	Qualitative	Caregivers with a child less than 2.5 years, facility-based providers, community health workers and district health services staff	Purposive	61	COVID-19 knowledge	Limited knowledge
								COVID-19 knowledge influences on health-seeking behaviour	Misconceptions, fear of COVID-19, structural changes, reduced income and rising cases of malnutrition
								Perceived barriers and challenges faced by facility-based providers	Lack of caregiver compliance with risk mitigation measures, caregiver fear of COVID-19 risk mitigation measures, lack of caregiver knowledge about COVID-19 and lack of supplies and protective equipment
								COVID-19 influences on families and communities	Increased food insecurity, increased prices, reduced livelihoods and reduced interactions with others
43	Bekele et al., 2022 (47)	Ethiopia (low-income)	To assess maternal, newborn and child health service utilisation during the first 6 months of the COVID-19 pandemic compared with prior to the pandemic	Mixed-methods	Quantitative component: users of the maternal, newborn and child health services Qualitative component: doctors, nurses, midwives and clinical officers	Not reported	Quantitative component: not reported Qualitative component: 31	Number of new family visits	Decreased (significant)
								Sick under 5 child visits	Decreased (significant)
								Number of antenatal and postnatal care visits	Decreased (not significant)
								Child immunisation	No difference
								Perceived barriers	Fear of disease transmission, economic hardship and transport service disruptions and restrictions
								Enablers of service utilisation	Communities’ decreased fear of COVID-19 and awareness-raising activities
44	Basnet et al., 2022 (68)	Nepal (lower middle-income)	to explore the experiences of maternity service providers during the pandemic, examining their perspectives from the point of individuals, families, society, institutions and government	Qualitative	Front-line health care providers	Purposive	10	Fear of COVID-19 at work	Causes of fear (transmission and uncertain outcomes), manifestations of fear (anxiety, irritability, loss of sleep, excessive handwashing and weight loss) and coping with fear.
								Challenges at work	Managing visiting crowding in hospital, staffing issues at work, issues with protective equipment at work and trainings and guidelines
								Changes at workplace and services	Changes in work infrastructures, changes in procedure and new protocols
								Factors influencing motivations to work	Enablers (professional responsibility to society) and impediments (no support and motivation from family and colleagues)
								Stigma due to COVID-19	Family/neighbours and institutions
								Impact on services	Decreased service utilisation and perceived poor quality of care
45	Thsehla et al., 2023 (75)	South Africa (upper middle-income)	To investigate the indirect effects of COVID-19 on maternal and child health in different geographical regions and relative wealth quintiles	Pre-post	Users of public maternal and child health services	Not reported	4,956	Child immunisation	Decreased
								Incidence and mortality due to child pneumonia, diarrhoea and severe acute malnutrition	Decreased
								First antenatal visits	Increased (not significant)
								Caesarean section delivery rates	Increased (not significant)
								Maternal mortality	Increased (not significant)

The results of the current review will be presented in two parts. Firstly, the impact on access to and delivery of maternal and child healthcare services will be presented in accordance with four groups of indicators. In the second part, the experiences of service users and healthcare professionals regarding the pandemic’s impact on access to and delivery of maternal and child healthcare services will be introduced.

### Impact on maternity service use and provision

#### Family planning services

In nine studies (34, 38, 41, 47, 49, 58, 67, 76, 77), the analysis showed interruptions in family planning services (76), a decrease in attendance of family planning visits (77), in the overall number of such visits (34, 41, 47, 49, 58, 67) and family planning acceptance rate (38) compared to the pre-COVID-19 levels. Although some authors observed a reduction in the number of new contraceptive acceptors (45) and difficulties accessing contraceptives (73), Tilahun and colleagues reported an increased contraceptive acceptance rate in Ethiopia (42). Three studies declared impaired abortion care during the pandemic in Ethiopia and India (38, 45, 66).

#### Antenatal and postnatal care coverage

Twenty-seven studies reported on antenatal care coverage during the pandemic using various metrics (34, 36–38, 42, 43, 47, 49–51, 56, 58, 59, 61–63, 65, 67, 70–78). Albeit no changes were made to the standard antenatal care protocol in the majority of settings, increased interruptions in antenatal care (76) and a decrease in antenatal care coverage (42), antenatal recruitment rate and prenatal visit completion rate (59), antenatal care registrations (62), number/proportion of antenatal care visits (34, 36–38, 47, 49, 56, 58, 61, 65, 67, 71, 72, 74, 75) and attendance (50, 51, 70, 77, 78) was noticed in most cases as compared to the pre-pandemic period. However, Pillay and colleagues (73) observed no difference in the number of first antenatal care visits in South Africa and Lydon and colleagues (43) detected an increased number of first antenatal visits and no difference in the number of fourth antenatal visits in Mozambique. No difficulties in accessing antenatal care were declared in one study originated from India (63). Due to the restrictions imposed during the COVID-19 pandemic, authors noticed a declining trend in the number of first routine laboratory tests (58), first and second trimester sonography (58, 66) and pregnant women receiving the second dose of tetanus toxoid vaccine during pregnancy (49). Furthermore, as per Burt and colleagues (37), the number of attendances for prevention of mother-to-child transmission of HIV dropped than stabilised in Uganda. A surge in the number of high-risk pregnancies was described in one study (56).

Although three studies highlighted reduced postnatal care (45, 67, 78), it was not universal as postnatal care coverage surged in Ethiopia (42).

#### Virtual care protocols

Despite the active promotion of virtual services during the pandemic, only one study from Cameroon reported an increase in the use of telemedicine services (57). According to Goyal and colleagues, just 3.6% of pregnant women living in India exploited teleconsultations amongst more than a thousand respondents (66).

#### Impact on institutional delivery

Included studies showed mixed results concerning institutional deliveries that comprise normal vaginal deliveries and caesarean sections. Even though eight studies highlighted a reduction in the number/proportion of institutional deliveries (36, 49–51, 56, 61, 62, 67, 71), six reports (38, 43, 63, 73–75) observed growth and two studies (34, 42) did not find any changes with respect to this indicator. The results varied depending on the setting in three multi-centred studies (64, 77, 78), making it difficult to provide a generalised conclusion. Home delivery rate rose based on the results of two studies originated from Mozambique and India (36, 62) and reduced in Ghana (48).

#### Birth outcomes

The impact of the COVID-19 pandemic on birth outcomes was reported in eight studies. Maternal mortality rates increased (64, 73, 75) and remained unaffected (37, 51) in three cases and two cases, respectively. A growth in stillbirth levels was observed in two studies (38, 63), and a decline was reported in one instance (51). Diverse results were obtained concerning the risk of adverse birth outcomes and obstetric complications (43, 64, 72).

### Impact on child service use and provision

Despite the fact that the rate of neonatal admissions increased in Uganda (37), its overall number declined in Ethiopia, Ghana and Guinea (34, 48, 64) as compared to the pre-pandemic period. Furthermore, a decrease in the number of consultations for sick children and emergency visits was observed in four different countries – Cameroon (57), Mexico (71), Ethiopia (34, 35) and South Africa (69). In the context of the COVID-19 pandemic, the level of early neonatal deaths increased in Uganda, Zimbabwe, Guinea and South Africa (37, 51, 64, 73). The majority of studies reported a fall in child immunisation levels (37, 38, 49, 71, 73, 75, 77–79). However, three studies highlighted that the number of children receiving scheduled vaccination increased in Ethiopia (38, 42) and India (62) and no changes with respect to this indicator were found in two studies from Ethiopia and Mozambique (39, 41).

### Experiences of service users and healthcare professionals

Identified concepts relevant to service users’ and healthcare professionals’ experiences regarding the impact of the COVID-19 pandemic on access to and delivery of maternal and child healthcare services were grouped into four main framework categories: the anxiety of not knowing (1), overwhelmed healthcare systems (2), challenges perceived by healthcare professionals (3) and difficulties perceived by service users (4). The respective sub-categories within each of these categories are reported in the section below. Illustrative quotations within each category are presented in Table 2.

**Table 2:** Illustrative quotations.

Categories and sub-categories (relevant studies)	Illustrative quotations
The anxiety of not knowing	
Limited knowledge (36, 46, 47)	“Media expresses it well; we know well it is also an infected person who can transmit it …” (47) “It can be transmitted through air/ breathing, shaking hands, kissing, contact with others and when face masks are not applied properly” (47) “…a very dangerous disease that can spread from person to person.” (46) “…a worldwide disease, which is very lethal, and communicable.” (46) “…respiratory disease that attacks the lungs, it causes coughs, muscle pains and diarrhoea.” (46) “…disease that came from China that attacks animals.” (46) “…it’s a flu, in which the person has a cough, headache, neck pain, feels cold and has fever.” (36) “…we have to wash our hands with water and soap or ashes.” (36) “… we have to use masks, whenever we go out!” (36) “…if the person travels to a country contaminated by Covid-19 he has to be quarantined for 14 days.” (36) “…everyone needs to use masks and maintain social distancing of 1.5 m.” (36)
Misconceptions and stigma (40, 42, 46, 47, 54, 61, 68)	“…disease that came from China that attacks animals.” (46) “…as my contemporaries started testing positive for COVID (…) the uncertainty around COVID further instilled more fear in me. (…) Later when I got posted in an isolation ward and saw many patients getting discharged. This allayed my fear to some extent…” (68) “I do not believe it exists, especially in our area. It might be real / exist in other areas/countries. They just suspect and take everyone into an isolation/quarantine center, but they are healthy and free of any signs and symptoms… “(47) “…I have never seen anyone with such a real problem in our area. We have heard about it on radio and TV, so I found it difficult to believe and I do not believe it is real” (47) “There are huge gaps, misconceptions, and challenges in practical preventive practices. They even perceived that the disease may not be real. Clients recovered from COVID-19 without any sign and symptom disseminated the information to the community and based on that the community misconceived that the virus might not be real from the beginning.” (47) “Everywhere you move, there is corona testing; you do not have an option for not to be tested and it is mandatory for everyone. The problem is that they test you in an open field where everybody can watch you. If, unfortunately, I become positive, I will be taken to hospital publicly, without keeping my secret.” (40) “I have witnessed that women who visit a health facility for any reason were considered to bring the virus into the community; thus, people refrain from meeting them.” (40) “Those who go to the hospital are victimized.” (54) “If they see me going to the hospital, they will badmouth about me.” (54) “The infected person lives a lonely life during isolation. I do not want to be a victim.” (54) “Recently, the neighboring lane was sealed. It has been only a week that the lane had opened. The entire family staying in front of us was COVID positive. We got so scared that neither did we go down nor let our children go down. We told the rest of the neighbors also to not go near them.” (61) “Gradually the community start adapts to the pandemic and their fear for the disease reduces time to time. Moreover, the community gets health information about coronavirus through health extension workers and through different media channels…” (42) “The health extension workers, health officers, and health facility workers were giving health education, using montarbo on every cluster of health centres.” (42)
Fear of contagion (36, 40, 42, 44, 46, 47, 54, 60, 61, 68)	“…I started washing hands frequently. (…) I had repetitive thoughts of washing my hands even during sleep…” (68) “You can have this risk [risk of contagion] at transport and at health facilities during service provision and from other clients/patients. That is the first fear.” (47) “Health professionals subjected to additional COVID-19 related tasks, patient flow decreased due to emerging concerns and fears of contracting the disease.” (47) “I have postponed my follow up at that time for fear of acquiring the disease from health professionals/health centres. The same is true for other clients in our area and some mothers have received their visit in private clinics as we perceived almost all staff were infected.” (47) “The community has been frightened of contracting the disease at the beginning.” (47) “At the beginning of Covid-19 occurrence, the community panicked and feared acquiring the disease.” (47) “At the beginning of coronavirus some people did not want to receive the services for fear of contracting the disease. So, client flow at that time has decreased.” (47) “The flux of patients is reduced; it may be because they fear coming to the hospital thinking that they might be contaminated here in the Nampula Central Hospital” (36) “I do not want to know my test results, because I cannot with stand the stress of being positive for corona virus. I have heard a story of many individuals who had attempted suicide.” (40) “I do not think that health facility environment is neat at this time. I doubt that they might not frequently clean surfaces, walls, chairs, and materials needed for treatment. If I go to health facility, I may contact with those unclean materials and get infected with the virus.” (40) “Health facilities give service for all clients coming from different areas; this results in overcrowding and makes it easier for corona transmission. Thus, rather than going to health facility, I prefer seeking advice from health extension worker.” (40) “Pregnant women who did not visit antenatal care could deliver safely without any problem, but if she gets infected with corona, she will be seriously ill and may not even survive. So, I would advise pregnant women not to visit health facility in this dangerous time.” (40) “How would one compare the benefit that the baby gets from antenatal care service utilization with the risk of getting corona by visiting health facility? In my opinion, the virus is much more serious than the problem that may occur to the baby from not using antenatal care service.” (40) “You see because we fear that hospital, they told us that there is a COVID-19 suspect. I went to the clinic and they injected me. … I am now worried.” (44) “I feared getting infected. I rather stay at home than get infected with the new virus.” (54) “I have heard a lot about the virus and I will not want to be a statistic.” (54) “…and I avoid going to the health centre, unless it is really urgent, because of this new infection.” (36) “We never went out as my daughter is very young. We never took her out because of so many cases of Corona infections.” (61) “When I was about to give birth, I felt so worried to go to the hospital. I was afraid that I might get COVID because we can get COVID in the hospital.” (60) “I’m just worried about my baby and family. I am still giving the services for the mothers, but I cut the duration. I mean I do not accept any patients after hours.” (60) “Generally the impact of COVID-19 in all health services especially in immunization service; parents were absent from the service area due to fear…” (42) “Right now, the entire community members have no fear or concern about acquiring the disease (…) we are not concerned about client decrement related to COVID-19. Specially after the 5 months state of emergency was lifted things are returned to pre-COVID time.” (47) “I feared going near the [patient’s] bed initially, but now my fear has slowly decreased after being posted to COVID hospital.” (68) “The caregivers reduced their consultations at the health facilities because of the fear of the unknown.” (46)
Overwhelmed healthcare services	
Insufficient staffing levels (36, 40, 44, 54, 60, 61, 63, 68)	“Although non-COVID wards have lesser patient flow, it is impossible to pool staff because our hospital has always had a chronic shortage of staff. In situations where pooling may be possible, the staff are reluctant to take up duties as they lack skills required for maternity services.” (68) “During this corona virus period, health care providers are facing huge challenges as staffs are assigned in different corona virus related tasks such as: isolation room, provision of health education, screening centres and etc. In this case, it is difficult for a single health care provider to provide antenatal care service alone and it would even be much more difficult on market days where most pregnant women often chose to visit antenatal care.” (40) “Of course, we see that in some places the there is a lot of prioritization on COVID-19 services. So we see that already especially when you go to the grass roots where we have very few health workers at the facility.” (44) “The fact that health workers who need to do [provide family planning services]; are the same health workers who are engaged in other tasks at the health facility. But also, as organizations, we had to shift. You cannot keep focusing on only family planning when people in the community are getting COVID-19.” (44) “There are not enough healthcare workers. It’s frustrating to wait for so long.” (54) “Last time I went but there was no healthcare worker to attend to patients.” (54) “Unavailability of healthcare providers.” (63) “The number of health professionals has decreased, and they leave early, so the waiting time has increased a bit.” (36) “… in the wards there is only one nurse per shift, and because of the pandemic if one gets sick, we will be forced to work every day to cover her!” (36) “First of all, there was only one person who was managing the hospital billing counter section. The queues were long, and one hospital staff was trying to manage the queue.” (61) “The midwife said that the vaccination officer would come, but he never came. So, I need to take him to Puskesmas.” (60)
Disrupted flows of commodities (pharmaceuticals and essential goods) (40, 42, 44, 60)	“Since the corona virus pandemic, we are facing a serious shortage of essential drugs and supplies like: alcohol, iron, face mask, and other personal protective equipment.” (40) “I do not think the health care facilities in this pandemic period have the necessary materials for providing antenatal care service....the Medias, the government, and everybody is saying corona, corona, corona...” (40) “In the last few months of my pregnancy, I did not get the Angel [multi-micronutrient supplement] anymore. The Posyandu was cancelled at that time. I came to the Puskesmas, but the midwife said there was no more stock.” (60) “It is difficult now to ask the pharmacy warehouse for a new supply. I have heard that the supply is very limited, and most of the supplements will expire soon.” (60) “In recent times there are shortages and interruptions of BCG [Bacille Calmette-Guérin] vaccines. We provide BCG vaccine for two weeks by sharing vaccine from other health facilities in the town but we have no BCG vaccine today onward…” (42) “Corona cannot be a reason for the difficulty to get inputs. Of course, there was a person who was transporting vaccination inputs from the woreda. After corona, he has not been willing to resume his usual task which is transporting the inputs.” (42) “I do not think there was too much impact on availability of commodities because we had cargo planes coming in; they were not stopped. National Medical Stores was open and I am not sure if really the delivery of National Medical Stores was affected by COVID-19. Also, I am not sure there was a great impact on our commodities but it was access to the commodities that was affected.” (44)
Decreased quality of care (40, 42, 46, 60, 61, 63, 68)	“This days everyone is talking about CORONA virus, and I do not think that healthcare providers have a time to treat pregnant women as usual. Thus, what is the point of visiting a health facility for antenatal care if you do not have enough time to be treated and advised?” (40) “Before COVID, we cared for our patients more closely with frequent conversations and patting on the back or holding hands to make them feel cared for was common. This was appreciated by the patient as well. Now due to the distancing rules, I feel we are providing inadequate mental health support to the patients in terms of them feeling adequately cared for.” (68) “Before the pandemic patients were keen to let them stay longer in hospital as they perceived better postnatal care at the hospital, but now they wish to get discharged as soon as they deliver which is also risky as the patient may not receive adequate postnatal care.” (68) “Before, the consultations were frequent or monthly, currently, consultations such as family planning, post-natal and pre-natal are done every 3 months.” (46) “Higher proportion of C-section deliveries especially in private health facilities.” (63) “Increased number of still-births.” (63) “Two women were asked to lie down on a 2.5 feet narrow delivery table in labour room. I was one of them. I was very scared of falling. Moreover, the toilet in the labour room was very dirty. The floor was blood-stained and the toilet had a foul stinking smell of urine.” (61) “I went there [the auxiliary Puskesmas] twice in the afternoon, but the Puskesmas was always closed. The registration counter was closed. It’s not like what I thought. It seems they closed [the service] earlier because of this Corona. Next visit, I tried to go to another Puskesmas, but the service was only until midday.” (60) “Because during this Corona the immunisation and [weight] measurement service was not there [Posyandu] anymore. […], I had to take my child to the Puskesmas for immunisation. But I did not go there, so I do not know his weight. The place is far away.” (60) “…it was difficult to give services on maternal and child health because there were direction and advice given not come at health institutions, due to this the performance now achieved is low. But on the immunization service had no negative impact on performance…” (42) “The accessibility and quality of the MCH [maternal and child health] service were highly cracked by the COVID-19 pandemic, i.e., poor quality with low accessibility of the usual health services…” (42) “The quality and coverage were affected by the pandemic. The service given was not adequate as the previous [services are given before COVID-19], the health workers were not actively involved in the routine health care services except emergency services, the community also not utilizing the health facility for MCH [maternal and child health] services…” (42) “All components (…) were very low during this year as compared to the last year with the same month. Home delivery was high during the pandemic as compared to before the pandemic (…). There is a facility that completely closes services like FP [family planning], ANC [antenatal care], and PNC [postnatal care]; except emergency. The services were totally/completely closed in the city area. Generally, there is low service utilization, accessibility and coverage; and a high number of home delivery due to the pandemic effect…” (42) “The Skilled delivery performance already low achievement before the COVID-19 occurrence, after COVID-19 the maternal health services follow-up activities were decreased too…” (42) “There is an impact on immunization, clients were worried about COVID−19 due to this they did not come to health institutions and missed different services.” (42) “Unavailability of ultrasound check-up.” (63) “Unavailability of laboratory services.” (63)
Transportation-related issues (40, 44, 47, 54, 61, 63, 68)	“…we have observed increased fresh and macerated stillbirth…this may be due to lack of transportation for timely arrival to the hospital, late admission of women at 41 to 42 weeks of pregnancy, and decreased antenatal visits. We could have saved more babies had they arrived earlier in their pregnancy.” (68) “Initially…mothers were staying at hospital unnecessarily due to absence of transportation/ambulance/.” (47) “Travel restrictions are also another reason for low client flow which is more pronounced amongst mothers from far kebeles.” (47) “Now, transportation cost is doubled. For this reason, I am forced to pay for two seats. Besides, it’s mandatory to wear a face mask unless they do not allow you to use the service. It is difficult for me to afford all those things where my income is decreased by the pandemic already.” (40) Even now with the restrictions on movements, that affected their [family planning users’] continuity of the product. So, for those people who were in lockdown, getting their new shot for Depo or oral contraception pills was difficult. This affected them in terms of continuity of access and utilisation of family planning methods.” (44) “There were clients coming to us [for family planning services], during the lockdown. They were accessing FP [family planning] services but not very much especially during the month of April and May–during that [total] lockdown.” (44) “Regarding access and utilization, we had challenges with health workers accessing facilities because the transport fares had been hiked. When transport fairs are hiked, that means we have challenges with them getting to work until of recent that the situation has certainly improved. However, in the beginning they worked with skeleton staff for the first three months of the pandemic.” (44) “Public transport is overcrowded, it is risky using it during this time.” (54) “Unavailability of transport to reach the health facilities.” (63) “I did not get an auto on time. Bus service was not operational. Due to this, I faced great difficulties during my pregnancy and at the time of delivery.” (61) “My delivery happened at home; the baby had come out. I could not make it to the hospital as I could not arrange for a mode of transport on time.” (61)
Challenges perceived by healthcare professionals	
Emotional toll (40, 42, 68)	“Whenever I talked with my neighbour, they advised me to take annual leave to stay home and take care of my child. However, being a government health worker, I was not allowed to take any type of leave during this period. This was so stressful for me to cope with.” (68) “One day I was in close contact with a patient, (…) providing cold sponging to a pregnant lady with a high fever. The ward was so busy that I could not find time to adequately wash my hands. Soon after that day, I tested positive for COVID.” (68) “…either having a separate operating room dedicated for COVID positive patients or operating on COVID positive patients at the separate COVID hospital would help reduce the exposure COVID amongst the staff.” (68) “I had undue pressure from my family to quit my job due to fear of COVID. My line manager provided a lot of support for my mental health and welfare. This gave me confidence to convince my family and continue my job.” (68) “My neighbours spread a rumour that I was COVID positive when I was home for 2 days. I felt stigmatized being labelled as COVID positive and people stared at me with suspicion and also ran away from me on the street. COVID has been used as a reason to stigmatise health workers. However many weeks later when one of them got infected with COVID and they needed my help. They started treating me nicely.” (68) “The discrimination towards health workers is so strong that they consider all health workers as a vehicle for COVID transmission in the community. Even my sister-in-law stopped talking to me. My children were not allowed to play in the public playground which is just in front of my house. This was hard for me to take on as my relatives were discriminating me, let alone the community people.” (68) “After working the whole day in the work place, at night I go home; imagine the risk I could bring to my family. Why would I take such a risk? Where the government is not even willing to pay a risk allowance, let alone arrange accommodation for staff. I have a family to support; I no longer have interest to work in this environment.” (40) “Generally speaking, the health workers feels fear of the pandemic, lacks PPE [personal protective equipment] and low commitment to serve before COVID were the major things which make their commitment under questions…” (42)
Shortage of personal protective equipment (40, 42, 68)	“As most people lost jobs, many hospital staff were the only bread earners of their family. In addition, as the Hospital did not provide adequate masks, we had to spend our own money to purchase the masks at extortionist prices to protect ourselves. Even if the hospital provided salary on time would be great motivation to me and my staff.” (68) “Our demand for PPE took long to go up the bureaucratic channel. When it did reach the right section of the hospital they were not clear about the procurement system in emergencies like the pandemic due to a lack of clarity of the administrative and financial regulations. Local philanthropic agencies finally donated some PPE to us.” (68) “If we take, for example, shortage of personal protective equipment, without them, the risk of transmitting the corona virus will be increased. To decrease the risk of transmission, we usually compromise the routine antenatal care service. For instance, we may not perform physical examination or draw blood, even if necessary.” (40) “The commitment of health workers was highly challenged and they are obligated to stop their routine activity due to frustration and lack of personal protective equipment. As any other community they have fear and frustration; lack of personal protective equipment’s…makes them fear…” (42)
Lack of service users’ compliance (36, 68)	“Managing extra people visiting the hospital was a real challenge for us. The number of security personnel was increased. This too did not work as the visitors verbally abused the security personnel and threatened to physically assault the personnel if they attempted to stop the visitors from entering the hospital. Furthermore, they spat all over the place when they were stopped. We try to do our best to minimize the number of visitors and motivate the visitors to comply with the hygiene measures. However, the compliance was poor as it seemed the visitors did not take COVID seriously so we could do nothing.” (68) “… patients and visitors do not wear masks and the cabin (private) rooms are always crowded with a lot of people visiting the patients. This is unsafe for everyone.” (68) “…the health professionals refuse to treat patients with no masks and that did not wash their hands!.” (36)
Difficulties perceived by service users	
Reduced/lost income and food insecurity (44, 46, 54, 60, 61)	“Before the coronavirus I used to be able to bring something for my daughter to eat. Now that the doors have closed during this time of coronavirus, my livelihood is very complicated. What I manage today is not 70% of what I used to get before the pandemic. This disease brought me some losses, life is so difficult in order to raise the children. For my daughter’s food am sacrificing at the moment.” (46) “In terms of nutrition the situation changed, the pandemic affected the whole economy of our community, markets were closed, very little was produced in the small farms, because people had movement restrictions... a lot of effort was done last year aiming at reducing [malnutrition] cases, but suddenly everything stopped. The children were the first to be affected by this situation.” (46) “…things have been difficult lately. For example, yesterday at home we slept without dinner because we had nothing to eat.” (46) “…there are days we sleep hungry, we have a house we used to rent but there are no clients now, there are days we go hungry.” (46) “Many of the caregivers lost their jobs and maybe businesses closed, because the market fairs were closed and that resulted in low income for many families and it became difficult for them to buy food to feed their children.” (46) “There is no money, only a few went to the fields to cultivate hence there is no produce, in the markets there are not a lot of things and the products prices have gone up.” (46) “She [daughter] does eat, but the prices of products have hiked a lot because of coronavirus [...]. Before yesterday, I went to buy Danone for my daughter and I saw that the price had change from 25 Meticais to 30 meticais and I was not able to buy. When I asked, they told me that coronavirus has blocked all the money.” (46) “Nowadays, when I go to the fields, at 04:00, I do not come back at 09:00 but at 06:00. This coronavirus has reduced our production, because we do not spend a lot of time like we used to before. Money today has disappeared and if we do not produce and sell, we will not have money to buy clothes for her.” (46) “There is lack of money nowadays and lack of food. The prices of food products have gone up and the men are complaining a lot that they are not able to buy things for children like before. There are no jobs.” (46) “Many having lost jobs during the lockdown, they are going to increase on poverty level. As a result, many young girls are going to get married to poor families and definitely poverty will also increase.” (44) “I shut my business during the corona time, now I do not have money at all.” (54) “My husband lost his job and we were depending on him. Now we have nothing.” (54) “For our child there are lots of expenses, which are difficult to bear after both my husband’s and mother’s jobs were lost. My breast milk is also not adequate because I am not able to have enough food.” (61) “Now, we are struggling because there is no income at all. […] I once went there [Puskesmas] when I was pregnant. Maybe about 1–2 km on walking. In there, I checked my pregnancy and paid around Rp25,000. So, I only came once because it was better to buy food than to pay for the Puskesmas. […] I was not strong enough to walk for 2 km away.” (60) “I could not take my son to the Puskesmas every month because I was afraid of paying something while I had no money. My husband does not have it [job], right? I only went there [Puskesmas] to bring my son for immunisation.” (60)
Increased put-of-pocket expenditure (54, 63, 68)	“The results for PCR test in our hospital takes up to 7 days. This creates an additional burden for patients who are admitted on a separate bed just to rule out COVID infection as the bed charges are ~10 USD/day. A patient recently came out negative for COVID who spent 7 days at the hospital was unable to pay the hospital charges of ~45 USD. As all expenses are out-of pocket, this is just so unfair to poor patients who have little means to afford it. Lack of adequate communication by staff and unclear administrative/finance regulations on the provision of free beds has led to this mishap.” (68) “Paying for services is very expensive. I could not afford it.” (54) “Services are not always cheap. You have to buy medicines all the time.” (54) “Higher expenses for the health services.” (63)
Healthcare providers’ unprofessional behaviour (40, 42, 54, 61, 68)	“As my neighbor told me, healthcare providers often use the same glove for different clients, and they do not use alcohol regularly; I think all they do care about is only for themselves. Some of them even move here and there but they do not change their gloves before toughing you.” (40) “I would not advice pregnant women to visit a health facility during this corona virus period. What I heard from those who visit a health facility is completely discouraging; health care providers often disgrace you and even insult you. Though, I do not blame them for doing so since they are taking a high risk; just think about working in the corona virus period? Hum... they have a family too.” (40) “Sometimes the harassment is too much to bear.” (54) “Healthcare workers are abusive and rude to the patients sometimes.” (54) “My previous experience was not pleasing. I will not be comfortable with the same healthcare provider.” (54) “I missed my last ultrasound during my pregnancy. Nurses used to avoid coming close. Doctors were not physically examining/touching. They used to observe from a distance, it was a very strange feeling. Nurses did not even talk properly.” (61) “It is difficult to explain in words what I have gone through during my pregnancy. I would not recommend others to go to that public hospital for delivery. Behaviour of hospital staff was unprofessional; I was not allowed to see the doctor. They told me to come in after two days.” (61) “The health workers were not giving the health services by keeping the professional ethics. The commitment to serve the community by keeping all the professional ethics was very low and compromised…” (42) “…The on-call physicians are reluctant to attend calls immediately and in most cases, they come only when called many times. This was not the case before COVID. Back then we had very prompt visits.” (68)

### The anxiety of not knowing

The anxiety of not knowing about COVID-19, particularly in the early stages of the pandemic, was a common and understandable response to the rapidly evolving situation. According to the participants, limited knowledge about the disease, misconceptions and stigma, and fear of contagion contributed to this anxiety.

#### Limited knowledge

Considering that COVID-19 was a completely new disease and there was little information available, participants demonstrated only basic and rather limited knowledge about its causes, symptoms, transmission and potential consequences (36, 46, 47). It was noted that COVID-19 is “a very dangerous disease” (46), which “can be transmitted through air/breathing, shaking hands, kissing, contact with others” (47). The essential measures, such as wearing a mask (36, 47), washing hands (36) and social distancing (36) were mentioned as helping to protect yourself and others from the disease.

#### Misconceptions and stigma

COVID-19 has not only been a health crisis but also a social and psychological challenge, leading to the rapid spread of misinformation (40, 42, 46, 47, 54, 61, 68). Misconceptions ranged from false information about its origin to conspiracy theories about its existence. In particular, participants believed that the virus “attacks animals” (46) and implied that it “may not be real” (47). Furthermore, it was reported that people diagnosed with COVID-19 or who had recovered from the virus were being victimised (54) and experienced discrimination as people tend to “badmouth” (54), “refrain from meeting them” (40) and “not go near them” (61). However, participants also highlighted that public awareness campaigns focusing on disseminating accurate information helped to address misconceptions and reduce stigma across different communities (42).

#### Fear of contagion

COVID-19 demonstrated rapid community transmission, resulting in widespread outbreaks across countries and continents. The exponential growth in cases has instilled fear of contagion in many individuals and communities (36, 40, 42, 44, 46, 47, 54, 60, 61, 68). Participants shared that healthcare facilities were considered as potential sources of COVID-19 transmission (36, 40, 42, 44, 47, 60); therefore, they tend to postpone or avoid general healthcare visits and antenatal care due to the “fear of acquiring the disease” (47). Participants also highlighted having anxious thoughts about the requirement to wash hands frequently (68) and the fear of testing positive for COVID-19 (40). Nevertheless, some participants underlined that “fear has slowly decreased” (68) when lockdowns were lifted (47).

### Overwhelmed healthcare services

During the COVID-19 pandemic, healthcare services in LMICs faced overwhelming issues due to the rapid and widespread transmission of the virus. A number of contributing factors were discussed, including insufficient staffing levels, disrupted flows of commodities, decreased quality of care, limited access due to transportation issues and patient flow fluctuations.

#### Insufficient staffing levels

Healthcare staff during the pandemic have been reassigned to the COVID-19 units (40, 44), leaving maternity and child services with fewer resources. Furthermore, participants highlighted that the pandemic had exacerbated the pre-existing “chronic shortage” (68) of healthcare staff, which resulted in longer waiting times (36, 54, 61). The increased risk of exposure to the virus amongst healthcare staff has also led to a significant reduction of available workforce, and there were cases where no healthcare workers were able to attend patients (54, 60).

#### Disrupted flow of commodities

Restrictions on travel, border closures, and lockdown measures during the COVID-19 pandemic disrupted the global chain of pharmaceuticals and essential goods (40, 42, 44, 60). Participants emphasised that they faced “a serious shortage of essential drugs and supplies” (40) and a limited supply of vaccines (42). Nevertheless, one participant noted incoming cargo planes continued to operate during the COVID-19 pandemic, maintaining the flow of essential commodities (44).

#### Decreased quality of care

Concerns regarding the quality of care were expressed by both service users and healthcare professionals (40, 42, 46, 60, 61, 63, 68). Service users experienced delays or cancellations of services (46), faced challenges in accessing healthcare facilities (42) and expressed concerns about infection control measures (61). Healthcare providers, in turn, highlighted that COVID-19 restrictions resulted in reduced personalised attention and care as “frequent conversations and patting on the back or holding hands” (68) were not possible. The availability of crucial services, such as ultrasound check-ups and laboratory services was limited (63). The preference of service users (mothers) to be discharged earlier after giving birth was also observed by healthcare providers, which undermined the quality of postnatal care (68). Moreover, healthcare professionals noted that the number of stillbirths and caesarian sections increased, whereas the proportion of skilled deliveries decreased in comparison to the pre-pandemic levels (42). According to participants, service users tend to miss their immunisation appointments due to safety concerns (42).

#### Transportation-related issues

A number of transportation-related issues impacting access to healthcare facilities became a significant challenge for many people across LMICs (40, 44, 47, 54, 61, 63, 68). Participants emphasised that public transportation systems reduced or suspended their operating services during the pandemic, which resulted in “late admission of women at 41 to 42 weeks of pregnancy” (68), absence of transportation options for patients from remote areas (47, 63) and cases where “delivery happened at home” (61). Notably, service users also “were staying at hospital unnecessarily” (47) due to the limitations of transportation services. Although seeking medical care was amongst the essential activities allowed during lockdowns, restrictions on movement worsened access to healthcare facilities (44). Furthermore, participants shared that “transport fares had been hiked” (44), leading to financial constraints and making it difficult for them to afford transportation (40, 44).

### Challenges perceived by healthcare professionals

Healthcare professionals experienced numerous challenges during the COVID-19 pandemic as they played a critical role in caring for patients and managing healthcare systems during a global health crisis. Some of the key challenges highlighted by participants included emotional toll, shortage of personal protective equipment and lack of service users’ compliance.

#### Emotional toll

Healthcare professionals had to cope with significant emotional stress and mental health challenges due to witnessing the suffering of patients (68) and fear for their own health and that of their families (40, 68). Participants also reported experiencing harassment and discrimination from members of the public who perceived them as “a vehicle for COVID transmission in the community” (68). Such hostile attitude towards healthcare professionals endangered their job motivation and commitment (42, 68).

#### Shortage of personal protective equipment

During the pandemic, there were widespread shortages of personal protective equipment (40, 42, 68), leading healthcare professionals to resort to buying it by themselves “at extortionist prices “(68) or relying on donations from philanthropic agencies (68). Inadequate access to protective equipment increased fear and risks of infection (42), which forced healthcare professionals to “compromise the routine antenatal care service” (40) by not performing physical or laboratory examinations.

#### Lack of service users’ compliance

Healthcare professionals encountered issues with service users’ compliance in following recommended health guidelines (36, 68). In particular, some individuals demonstrated aggressive behaviour by threatening “to physically assault the personnel if they attempted to stop the visitors from entering the hospital” (68) or were reluctant to wear masks or practise social distancing (36, 68).

### Difficulties perceived by service users

Participants of the study shared difficulties that affected their healthcare experiences and overall well-being. Reduced/lost income and food insecurity, increased out-of-pocket expenditure and healthcare professionals’ unprofessional behaviour were reported as major ones.

#### Reduced/lost income and food insecurity

The economic impact of the COVID-19 pandemic on LMICs has been significant and exacerbated existing vulnerabilities. Many businesses had to shut down or reduce operations, resulting in widespread job losses and furloughs. Participants noticed that “many of the caregivers lost their jobs” (46) and they are struggling “because there is no income at all” (60). Loss of livelihoods, food price inflation, and disruptions to agricultural activities made it challenging to meet basic food needs (44, 46, 54, 60, 61). Participants admitted that “it was better to buy food than to pay” (60) for healthcare services.

#### Increased Out-of-pocket expenditure

Participants highlighted that increased out-of-pocket expenditure for healthcare services during the pandemic had considerable implications for individuals and families with limited financial resources (54, 63, 68). High healthcare costs resulted in avoided medical care and heightened health risks (54, 68).

#### Healthcare providers’ unprofessional behaviour

Service users admitted to facing numerous cases of healthcare providers’ unprofessional behaviour. Unprofessional behaviour involved a lack of empathy and compassion for patients and their families during such challenging times (42). Patients described their experience as “completely discouraging” (40) and “not pleasing” (54) because healthcare professionals were “abusive and rude” (54). Inappropriate adherence to infection control measures, such as using “the same glove for different clients” (40) and reluctance to physically examine patients (61) and attend calls (68) was also mentioned as examples of unprofessional behaviour.

## Discussion

### Main findings

Based on the findings from 45 unique studies conducted across 28 LMICs, the current review suggests that the COVID-19 pandemic disrupted access to and delivery of maternal and child services. In particular, the number of family planning visits, antenatal and postnatal care visits, consultations for sick children, paediatric emergency visits and child immunisation levels decreased as compared to the pre-pandemic levels in the majority of included studies. In contrast, a rise was observed in the number of neonatal admissions and early neonatal deaths. Inconclusive results were acquired concerning the number of institutional deliveries, adverse birth outcomes and obstetric complications.

The analytical framework that comprised four main categories of the anxiety of not knowing (1), overwhelmed healthcare systems (2), challenges perceived by healthcare professionals (3) and difficulties perceived by service users (4) was developed based on the concepts that emerged from included studies. Participants shared that limited knowledge about COVID-19, along with misconceptions and fear of contagion, led to people avoiding seeking healthcare. Unsurprisingly, participants also highlighted that maternity and child healthcare services were disrupted by significant challenges presented during the pandemic, including insufficient staffing levels, disrupted flow of commodities, decreased quality of care and transportation-related issues. On a personal level, healthcare professionals have reported experiencing a profound emotional toll, shortage of personal protective equipment and lack of service users’ compliance in the context of high workload due to the constant demand for healthcare services. Service users, in turn, have reported that issues, such as reduced/lost income and food insecurity, increased out-of-pocket expenditure and healthcare professionals’ unprofessional behaviour affected their ability to receive timely care. Identified main categories and respective sub-categories relevant to service users’ and healthcare professionals’ experiences regarding the impact of the COVID-19 pandemic on access to and delivery of maternal and child healthcare services were closely linked and largely overlapped. For example, healthcare professionals and service users shared the anxiety of not knowing about the novel coronavirus, which may have led to decreased quality of provided care and a lack of patient compliance. Overwhelmed healthcare services, in turn, have contributed to an enormous emotional toll amongst healthcare professionals and may have been a reason for their unprofessional behaviour noted by service users.

### Strengths and limitations

To our knowledge, this is the first systematic review aiming to determine the impact of the COVID-19 pandemic on access to and delivery of maternal and child healthcare services in LMICs. A further strength is that the review used a comprehensive approach, searching through studies from all LMICs, which allowed to include data from different countries and cultural backgrounds. However, this approach presented several limitations. Firstly, due to the heterogeneity of included studies, the variety of reported outcomes and their limited quality, it was not possible to conduct a meta-analysis; therefore, the final interpretation of quantitative data was made based on descriptive-analytical procedures. Such considerable heterogeneity also suggests that the findings of the current review should be interpreted with caution. Secondly, although it was possible to extract general concepts relevant to service users’ and healthcare professionals’ experiences regarding the impact of the COVID-19 pandemic on access to and delivery of maternal and child healthcare services, there is not enough evidence to assess whether these apply to all LMICs. There might be regional or clinical characteristics that have not been identified in this review. Finally, the comparability of findings across the included studies may be limited due to wide variability in periods (first wave, lockdown, second wave, etc.) when studies were conducted, local public health messaging to which people were exposed, national-specific circumstances and cultural differences. Also, the majority of studies were focused on African countries, which made it challenging to generalise any conclusions about LMICs.

### Comparison with literature from high-income countries

Similar to the findings of the current review, disruptions in the antenatal and postnatal care coverage were observed by numerous studies from HICs. In particular, a decrease in the number of antenatal visits (80–87), prenatal genetic diagnostic procedures (88) and performed obstetric ultrasound scans (89, 90) was reported alongside reduced postnatal care (91) in the United States, United Kingdom, Italy, Belgium and Saudi Arabia. These informal comparisons might suggest that healthcare professionals and patients from both HICs and LMICs perceived similar challenges during the COVID-19 pandemic. However, no change in antenatal care attendance (92, 93) and an increased number of the first-trimester prenatal screenings (94) were determined in the United States and Italy, respectively, highlighting inconsistencies in the obtained results due to wide variability of possible influencing factors. Although the results from LMICs were inconclusive regarding obstetric complications, the data from the United States and Israel suggests a decline in the number of obstetric emergency department visits (95, 96) and obstetric hospitalisations (97). This underlines the need for detailed analyses and the consideration of specific contexts in order to provide firm conclusions.

According to the report by the World Health Organization, disruption in the delivery of maternal and child health services was caused by two main reasons: “changes in demand and patient behaviour” and “changes in health-care supply” (98). This corroborates the findings of the current review that patients’ healthcare-seeking behaviour considerably changed due to the fear of contagion and misconceptions about COVID-19. Several studies from HICs support this statement by reporting that patients tend to cancel or ignore their appointments due to the risk of COVID-19 exposure and expressed a preference for shorter hospital stays after giving birth (80, 99–103). Reduced income and food insecurity during the pandemic have also played a significant role in influencing healthcare-seeking behaviour in LMICs. It seems predictable that individuals may prioritise meeting basic needs over seeking healthcare in situations of severe economic hardship, particularly in resource-scarce settings. Such changed maternity care-seeking behaviour determined in the current review might need to be perceived as potentially contributing to poorer birth outcomes. Even though the findings of the review were mixed, it appears reasonable to assume that not attending antenatal care visits, for example, might be associated with poorer pregnancy outcomes.

The alterations in the healthcare-seeking behaviour happened in the context of overwhelmed healthcare systems, leading to challenges to the quality of delivered care. It is important to note that increased use of telemedicine has only rarely been mentioned in studies of LMICs (47) albeit it was extensively discussed across studies conducted in HICs (93, 104–106). This indicates that whilst antenatal and postnatal care has transformed into a hybrid mode in HICs, minimising the pandemics’ impact on maternity and child care, antenatal and postnatal care services in LMICs faced often unavoidable ramifications. The COVID-19 pandemic has once again demonstrated inequalities between societies and regions as the majority of technological benefits were available to financially secure patients from HICs.

### Implications for research and practice

In order to generate clear directives for improvements, future research should aim at creating a set of indicators, allowing for direct cross-country comparisons and enabling to evaluate the scale of maternal and child healthcare disruptions during the pandemic. Moreover, future research studies may need to perform a comprehensive analysis of actions undertaken throughout the COVID-19 pandemic, which can be used to develop a healthcare delivery plan for emergency situations. This may help to build resilient healthcare systems in low-resource settings.

By considering the findings of the present review, future healthcare policies might need to prioritise helping LMICs adopt telemedicine into their healthcare systems. This would require a comprehensive approach that involves collaboration between governments, healthcare providers, technology developers and communities as a range of major challenges, such as limited access to reliable internet connectivity, lack of technical resources, electricity outrages, absence of clear regulations governing telemedicine, data privacy concerns, digital illiteracy and cultural resistance to change should be addressed. Supporting healthcare professionals after the COVID-19 pandemic to address the physical, mental and emotional toll they have experienced is also crucial to ensure a sustainable and resilient healthcare workforce. Providing regular counselling sessions, implementing flexible scheduling options, offering opportunities for continuing education and developing resilience-building programmes might help healthcare professionals recover from the impact of the pandemic. Finally, establishing collaboration and sharing experiences amongst countries seems essential to prepare maternal and child health services for future pandemics and improve global health outcomes. Facilitating collaborative research projects, offering cross-border training and knowledge exchange, empowering communities to implement community-led interventions and promoting culturally sensitive approaches may assist in enhancing pandemic preparedness.

## Conclusion

The current review has identified that COVID-19 has presented an unparalleled challenge to maternal and child health services in LMICs by disrupting family planning services, antenatal and postnatal care coverage, and emergency and routine child services. However, generalised conclusions are tentative due to the heterogeneity and inconsistent quality of the included studies. Investigating the pandemic’s impact is crucial to mitigate its negative consequences on women and children worldwide and prepare healthcare systems for future resurgences of COVID-19 and potential challenges beyond.

## Data availability statement

The original contributions presented in the study are included in the article/Supplementary material, further inquiries can be directed to the corresponding author.

## Author contributions

AK: Conceptualization, Formal analysis, Investigation, Methodology, Writing – original draft, Writing – review & editing. M-AO: Data curation, Investigation, Methodology, Writing – review & editing. MN: Data curation, Formal analysis, Writing – review & editing. AS-S: Conceptualization, Formal analysis, Funding acquisition, Methodology, Supervision, Writing – review & editing.
