# Supplementary material for: Impact of the COVID-19 pandemic on access to and delivery of maternal and child healthcare services in low-and middle-income countries: a systematic review of the literature
Source: Front Public Health. 2024 Apr 8;12:1346268. doi: 10.3389/fpubh.2024.1346268 (PMC11036866; doi:10.3389/fpubh.2024.1346268)
Supplement: Supplementary file 1 [file Data_Sheet_1.docx]

**Supplementary file 1. Search terms.**

impact OR effect OR influence OR “consequence*” OR significance

AND

COVID-19 OR COVID OR COVID19 OR coronavirus OR “SARS-CoV-2” OR “2019-nCoV”

AND

“maternal healthcare services” OR “reproductive healthcare services” OR “child healthcare services” OR “reproductive health” OR “maternal health” OR “child health” OR “newborn health” OR “family planning” OR pregnancy OR “birth control” OR abortion

AND

“low- and middle-income countries” OR LMICs OR “low and middle income countries” OR Afghanistan OR “Gambia” OR Niger OR Benin OR Guinea OR Rwanda OR “Burkina Faso” OR “Sierra Leone” OR Burundi OR Haiti OR Somalia OR Cambodia OR Korea OR “South Sudan” OR “Central African Republic” OR Liberia OR Tanzania OR Chad OR Madagascar OR Togo OR Comoros OR Malawi OR Uganda OR Congo OR Mali OR Zimbabwe OR Eritrea OR Mozambique OR Ethiopia OR Nepal OR Armenia OR Indonesia OR Samoa OR Bangladesh OR Kenya OR Bhutan OR Kiribati OR Senegal OR Bolivia OR Kosovo OR “Solomon Islands” OR “Cabo Verde” OR Kyrgyzstan OR “Sri Lanka” OR Cameroon OR Sudan OR Congo OR Lesotho OR Swaziland OR “Cote divoire” OR Mauritania OR “Syrian Arab Republic” OR Djibouti OR Micronesia OR Tajikistan OR Egypt OR Moldova OR Timor-Leste OR “El Salvador” OR Morocco OR Ukraine OR Georgia OR Myanmar OR Uzbekistan OR Ghana OR Nicaragua OR Vanuatu OR Guatemala OR Nigeria OR Vietnam OR Guyana OR Pakistan OR “West Bank and Gaza” OR Honduras OR “Papua New Guinea” OR Yemen OR India OR Philippines OR Zambia OR Albania OR Fiji OR Namibia OR Algeria OR Gabon OR Palau OR “American Samoa” OR Grenada OR Panama OR Angola OR Iran OR Paraguay OR Azerbaijan OR Iraq OR Peru OR Belarus OR Jamaica OR Romania OR Belize OR Jordan OR Serbia OR “Bosnia and Herzegovina” OR Kazakhstan OR “South Africa” OR Botswana OR Lebanon OR Brazil OR Libya OR Bulgaria OR Macedonia OR Suriname OR China OR Malaysia OR Thailand OR Colombia OR Maldives OR Tonga OR “Costa Rica” OR “Marshall Islands” OR Tunisia OR Cuba OR Mauritius OR Turkey OR Dominica OR Mexico OR Turkmenistan OR “Dominican Republic” OR Mongolia OR Tuvalu OR Ecuador OR Montenegro
