# Supplementary material for: Impact of the COVID-19 pandemic on access to and delivery of maternal and child healthcare services in low-and middle-income countries: a systematic review of the literature
Source: Front Public Health. 2024 Apr 8;12:1346268. doi: 10.3389/fpubh.2024.1346268 (PMC11036866; doi:10.3389/fpubh.2024.1346268)
Supplement: Supplementary file 2 [file Data_Sheet_2.docx]

**Supplementary file 2. Search strategy.**

1. Pubmed – conducted on 15^th^ of October – **211 results**

| **Search** | **Actions** | **Details** | **Query** | **Results** | **Time** |
| --- | --- | --- | --- | --- | --- |
| #5 |  |  | Search: **#1 AND #2 AND #3 AND #4** | [211](https://pubmed.ncbi.nlm.nih.gov/?term=%231+AND+%232+AND+%233+AND+%234&sort=) | 03:38:54 |
| #4 |  |  | Search: **”low-[Title/Abstract] AND middle-income countries”[Title/Abstract] OR LMICs[Title/Abstract] OR “low[Title/Abstract] AND middle income countries”[Title/Abstract] OR Afghanistan[Title/Abstract] OR “Gambia”[Title/Abstract] OR Niger[Title/Abstract] OR Benin[Title/Abstract] OR Guinea[Title/Abstract] OR Rwanda[Title/Abstract] OR “Burkina Faso”[Title/Abstract] OR “Sierra Leone”[Title/Abstract] OR Burundi[Title/Abstract] OR Haiti[Title/Abstract] OR Somalia[Title/Abstract] OR Cambodia[Title/Abstract] OR Korea[Title/Abstract] OR “South Sudan”[Title/Abstract] OR “Central African Republic”[Title/Abstract] OR Liberia[Title/Abstract] OR Tanzania[Title/Abstract] OR Chad[Title/Abstract] OR Madagascar[Title/Abstract] OR Togo[Title/Abstract] OR Comoros[Title/Abstract] OR Malawi[Title/Abstract] OR Uganda[Title/Abstract] OR Congo[Title/Abstract] OR Mali[Title/Abstract] OR Zimbabwe[Title/Abstract] OR Eritrea[Title/Abstract] OR Mozambique[Title/Abstract] OR Ethiopia[Title/Abstract] OR Nepal[Title/Abstract] OR Armenia[Title/Abstract] OR Indonesia[Title/Abstract] OR Samoa[Title/Abstract] OR Bangladesh[Title/Abstract] OR Kenya[Title/Abstract] OR Bhutan[Title/Abstract] OR Kiribati[Title/Abstract] OR Senegal[Title/Abstract] OR Bolivia[Title/Abstract] OR Kosovo[Title/Abstract] OR “Solomon Islands”[Title/Abstract] OR “Cabo Verde”[Title/Abstract] OR Kyrgyzstan[Title/Abstract] OR “Sri Lanka”[Title/Abstract] OR Cameroon[Title/Abstract] OR Sudan[Title/Abstract] OR Congo[Title/Abstract] OR Lesotho[Title/Abstract] OR Swaziland[Title/Abstract] OR “Cote divoire”[Title/Abstract] OR Mauritania[Title/Abstract] OR “Syrian Arab Republic”[Title/Abstract] OR Djibouti[Title/Abstract] OR Micronesia[Title/Abstract] OR Tajikistan[Title/Abstract] OR Egypt[Title/Abstract] OR Moldova[Title/Abstract] OR Timor-Leste[Title/Abstract] OR “El Salvador”[Title/Abstract] OR Morocco[Title/Abstract] OR Ukraine[Title/Abstract] OR Georgia[Title/Abstract] OR Myanmar[Title/Abstract] OR Uzbekistan[Title/Abstract] OR Ghana[Title/Abstract] OR Nicaragua[Title/Abstract] OR Vanuatu[Title/Abstract] OR Guatemala[Title/Abstract] OR Nigeria[Title/Abstract] OR Vietnam[Title/Abstract] OR Guyana[Title/Abstract] OR Pakistan[Title/Abstract] OR “West Bank[Title/Abstract] AND Gaza”[Title/Abstract] OR Honduras[Title/Abstract] OR “Papua New Guinea”[Title/Abstract] OR Yemen[Title/Abstract] OR India[Title/Abstract] OR Philippines[Title/Abstract] OR Zambia[Title/Abstract] OR Albania[Title/Abstract] OR Fiji[Title/Abstract] OR Namibia[Title/Abstract] OR Algeria[Title/Abstract] OR Gabon[Title/Abstract] OR Palau[Title/Abstract] OR “American Samoa”[Title/Abstract] OR Grenada[Title/Abstract] OR Panama[Title/Abstract] OR Angola[Title/Abstract] OR Iran[Title/Abstract] OR Paraguay[Title/Abstract] OR Azerbaijan[Title/Abstract] OR Iraq[Title/Abstract] OR Peru[Title/Abstract] OR Belarus[Title/Abstract] OR Jamaica[Title/Abstract] OR Romania[Title/Abstract] OR Belize[Title/Abstract] OR Jordan[Title/Abstract] OR Serbia[Title/Abstract] OR “Bosnia[Title/Abstract] AND Herzegovina”[Title/Abstract] OR Kazakhstan[Title/Abstract] OR “South Africa”[Title/Abstract] OR Botswana[Title/Abstract] OR Lebanon[Title/Abstract] OR “St. Lucia”[Title/Abstract] OR Brazil[Title/Abstract] OR Libya[Title/Abstract] OR “St. Vincent[Title/Abstract] AND the Grenadines”[Title/Abstract] OR Bulgaria[Title/Abstract] OR Macedonia[Title/Abstract] OR Suriname[Title/Abstract] OR China[Title/Abstract] OR Malaysia[Title/Abstract] OR Thailand[Title/Abstract] OR Colombia[Title/Abstract] OR Maldives[Title/Abstract] OR Tonga[Title/Abstract] OR “Costa Rica”[Title/Abstract] OR “Marshall Islands”[Title/Abstract] OR Tunisia[Title/Abstract] OR Cuba[Title/Abstract] OR Mauritius[Title/Abstract] OR Turkey[Title/Abstract] OR Dominica[Title/Abstract] OR Mexico[Title/Abstract] OR Turkmenistan[Title/Abstract] OR “Dominican Republic”[Title/Abstract] OR Mongolia[Title/Abstract] OR Tuvalu[Title/Abstract] OR Ecuador[Title/Abstract] OR Montenegro[Title/Abstract]** | [1,213,911](https://pubmed.ncbi.nlm.nih.gov/?term=longquerya750a5dc63cb4e6c50b0&sort=) | 03:38:03 |
| #3 |  |  | Search: **”maternal healthcare services”[Title/Abstract] OR “reproductive healthcare services”[Title/Abstract] OR “child healthcare services”[Title/Abstract] OR “reproductive health”[Title/Abstract] OR “maternal health”[Title/Abstract] OR “child health”[Title/Abstract] OR “newborn health”[Title/Abstract] OR “family planning”[Title/Abstract] OR pregnancy[Title/Abstract] OR “birth control”[Title/Abstract] OR abortion[Title/Abstract]** | [531,983](https://pubmed.ncbi.nlm.nih.gov/?term=%E2%80%9Cmaternal+healthcare+services%E2%80%9D%5BTitle%2FAbstract%5D+OR+%E2%80%9Creproductive+healthcare+services%E2%80%9D%5BTitle%2FAbstract%5D+OR+%E2%80%9Cchild+healthcare+services%E2%80%9D%5BTitle%2FAbstract%5D+OR+%E2%80%9Creproductive+health%E2%80%9D%5BTitle%2FAbstract%5D+OR+%E2%80%9Cmaternal+health%E2%80%9D%5BTitle%2FAbstract%5D+OR+%E2%80%9Cchild+health%E2%80%9D%5BTitle%2FAbstract%5D+OR+%E2%80%9Cnewborn+health%E2%80%9D%5BTitle%2FAbstract%5D+OR+%E2%80%9Cfamily+planning%E2%80%9D%5BTitle%2FAbstract%5D+OR+pregnancy%5BTitle%2FAbstract%5D+OR+%E2%80%9Cbirth+control%E2%80%9D%5BTitle%2FAbstract%5D+OR+abortion%5BTitle%2FAbstract%5D&sort=) | 03:37:29 |
| #2 |  |  | Search: **COVID-19[Title/Abstract] OR COVID[Title/Abstract] OR COVID19[Title/Abstract] OR coronavirus[Title/Abstract] OR “SARS-CoV-2”[Title/Abstract] OR “2019-nCoV”[Title/Abstract]** | [191,489](https://pubmed.ncbi.nlm.nih.gov/?term=COVID-19%5BTitle%2FAbstract%5D+OR+COVID%5BTitle%2FAbstract%5D+OR+COVID19%5BTitle%2FAbstract%5D+OR+coronavirus%5BTitle%2FAbstract%5D+OR+%E2%80%9CSARS-CoV-2%E2%80%9D%5BTitle%2FAbstract%5D+OR+%E2%80%9C2019-nCoV%E2%80%9D%5BTitle%2FAbstract%5D&sort=) | 03:37:13 |
| #1 |  |  | Search: **impact[Title/Abstract] OR effect[Title/Abstract] OR influence[Title/Abstract] OR “consequence*”[Title/Abstract] OR significance[Title/Abstract]** | [5,982,761](https://pubmed.ncbi.nlm.nih.gov/?term=impact%5BTitle%2FAbstract%5D+OR+effect%5BTitle%2FAbstract%5D+OR+influence%5BTitle%2FAbstract%5D+OR+%E2%80%9Cconsequence%2A%E2%80%9D%5BTitle%2FAbstract%5D+OR+significance%5BTitle%2FAbstract%5D&sort=) | 03:36:55 |

1. Scopus – conducted on 15^th^ of October 2021 – **467 results**

5

( TITLE-ABS-KEY ( impact  OR  effect  OR  influence  OR  ”consequence*”  OR  significance ) )  AND  ( TITLE-ABS-KEY ( covid-19  OR  covid  OR  covid19  OR  coronavirus  OR  ”SARS-CoV-2”  OR  ”2019-nCoV” ) )  AND  ( TITLE-ABS-KEY ( ”maternal healthcare services”  OR  ”reproductive healthcare services”  OR  ”child healthcare services”  OR  ”reproductive health”  OR  ”maternal health”  OR  ”child health”  OR  ”newborn health”  OR  ”family planning”  OR  pregnancy  OR  ”birth control”  OR  abortion ) )  AND  ( TITLE-ABS-KEY ( ”low- and middle-income countries”  OR  lmics  OR  ”low and middle income countries”  OR  afghanistan  OR  ”Gambia”  OR  niger  OR  benin  OR  guinea  OR  rwanda  OR  ”Burkina Faso”  OR  ”Sierra Leone”  OR  burundi  OR  haiti  OR  somalia  OR  cambodia  OR  korea  OR  ”South Sudan”  OR  ”Central African Republic”  OR  liberia  OR  tanzania  OR  chad  OR  madagascar  OR  togo  OR  comoros  OR  malawi  OR  uganda  OR  congo  OR  mali  OR  zimbabwe  OR  eritrea  OR  mozambique  OR  ethiopia  OR  nepal  OR  armenia  OR  indonesia  OR  samoa  OR  bangladesh  OR  kenya  OR  bhutan  OR  kiribati  OR  senegal  OR  bolivia  OR  kosovo  OR  ”Solomon Islands”  OR  ”Cabo Verde”  OR  kyrgyzstan  OR  ”Sri Lanka”  OR  cameroon  OR  sudan  OR  congo  OR  lesotho  OR  swaziland  OR  ”Cote divoire”  OR  mauritania  OR  ”Syrian Arab Republic”  OR  djibouti  OR  micronesia  OR  tajikistan  OR  egypt  OR  moldova  OR  timor-leste  OR  ”El Salvador”  OR  morocco  OR  ukraine  OR  georgia  OR  myanmar  OR  uzbekistan  OR  ghana  OR  nicaragua  OR  vanuatu  OR  guatemala  OR  nigeria  OR  vietnam  OR  guyana  OR  pakistan  OR  ”West Bank and Gaza”  OR  honduras  OR  ”Papua New Guinea”  OR  yemen  OR  india  OR  philippines  OR  zambia  OR  albania  OR  fiji  OR  namibia  OR  algeria  OR  gabon  OR  palau  OR  ”American Samoa”  OR  grenada  OR  panama  OR  angola  OR  iran  OR  paraguay  OR  azerbaijan  OR  iraq  OR  peru  OR  belarus  OR  jamaica  OR  romania  OR  belize  OR  jordan  OR  serbia  OR  ”Bosnia and Herzegovina”  OR  kazakhstan  OR  ”South Africa”  OR  botswana  OR  lebanon  OR  ”St. Lucia”  OR  brazil  OR  libya  OR  ”St. Vincent and the Grenadines”  OR  bulgaria  OR  macedonia  OR  suriname  OR  china  OR  malaysia  OR  thailand  OR  colombia  OR  maldives  OR  tonga  OR  ”Costa Rica”  OR  ”Marshall Islands”  OR  tunisia  OR  cuba  OR  mauritius  OR  turkey  OR  dominica  OR  mexico  OR  turkmenistan  OR  ”Dominican Republic”  OR  mongolia  OR  tuvalu  OR  ecuador  OR  montenegro ) ) View Less

[467 document results](https://www-scopus-com.ezproxy.library.qmul.ac.uk/search/history/results.uri?origin=searchhistory&shid=5)

4

TITLE-ABS-KEY ( ”low- and middle-income countries”  OR  lmics  OR  ”low and middle income countries”  OR  afghanistan  OR  ”Gambia”  OR  niger  OR  benin  OR  guinea  OR  rwanda  OR  ”Burkina Faso”  OR  ”Sierra Leone”  OR  burundi  OR  haiti  OR  somalia  OR  cambodia  OR  korea  OR  ”South Sudan”  OR  ”Central African Republic”  OR  liberia  OR  tanzania  OR  chad  OR  madagascar  OR  togo  OR  comoros  OR  malawi  OR  uganda  OR  congo  OR  mali  OR  zimbabwe  OR  eritrea  OR  mozambique  OR  ethiopia  OR  nepal  OR  armenia  OR  indonesia  OR  samoa  OR  bangladesh  OR  kenya  OR  bhutan  OR  kiribati  OR  senegal  OR  bolivia  OR  kosovo  OR  ”Solomon Islands”  OR  ”Cabo Verde”  OR  kyrgyzstan  OR  ”Sri Lanka”  OR  cameroon  OR  sudan  OR  congo  OR  lesotho  OR  swaziland  OR  ”Cote divoire”  OR  mauritania  OR  ”Syrian Arab Republic”  OR  djibouti  OR  micronesia  OR  tajikistan  OR  egypt  OR  moldova  OR  timor-leste  OR  ”El Salvador”  OR  morocco  OR  ukraine  OR  georgia  OR  myanmar  OR  uzbekistan  OR  ghana  OR  nicaragua  OR  vanuatu  OR  guatemala  OR  nigeria  OR  vietnam  OR  guyana  OR  pakistan  OR  ”West Bank and Gaza”  OR  honduras  OR  ”Papua New Guinea”  OR  yemen  OR  india  OR  philippines  OR  zambia  OR  albania  OR  fiji  OR  namibia  OR  algeria  OR  gabon  OR  palau  OR  ”American Samoa”  OR  grenada  OR  panama  OR  angola  OR  iran  OR  paraguay  OR  azerbaijan  OR  iraq  OR  peru  OR  belarus  OR  jamaica  OR  romania  OR  belize  OR  jordan  OR  serbia  OR  ”Bosnia and Herzegovina”  OR  kazakhstan  OR  ”South Africa”  OR  botswana  OR  lebanon  OR  ”St. Lucia”  OR  brazil  OR  libya  OR  ”St. Vincent and the Grenadines”  OR  bulgaria  OR  macedonia  OR  suriname  OR  china  OR  malaysia  OR  thailand  OR  colombia  OR  maldives  OR  tonga  OR  ”Costa Rica”  OR  ”Marshall Islands”  OR  tunisia  OR  cuba  OR  mauritius  OR  turkey  OR  dominica  OR  mexico  OR  turkmenistan  OR  ”Dominican Republic”  OR  mongolia  OR  tuvalu  OR  ecuador  OR  montenegro ) View Less

[4,643,913 document results](https://www-scopus-com.ezproxy.library.qmul.ac.uk/search/history/results.uri?origin=searchhistory&shid=4)

3

TITLE-ABS-KEY ( ”maternal healthcare services”  OR  ”reproductive healthcare services”  OR  ”child healthcare services”  OR  ”reproductive health”  OR  ”maternal health”  OR  ”child health”  OR  ”newborn health”  OR  ”family planning”  OR  pregnancy  OR  ”birth control”  OR  abortion )

[1,310,559 document results](https://www-scopus-com.ezproxy.library.qmul.ac.uk/search/history/results.uri?origin=searchhistory&shid=3)

2

TITLE-ABS-KEY ( covid-19  OR  covid  OR  covid19  OR  coronavirus  OR  ”SARS-CoV-2”  OR  ”2019-nCoV” )

[252,437 document results](https://www-scopus-com.ezproxy.library.qmul.ac.uk/search/history/results.uri?origin=searchhistory&shid=2)

1

TITLE-ABS-KEY ( impact  OR  effect  OR  influence  OR  ”consequence*”  OR  significance )

[21,447,649 document results](https://www-scopus-com.ezproxy.library.qmul.ac.uk/search/history/results.uri?origin=searchhistory&shid=1)

1. Embase - conducted on 15^th^ of October 2021 – **1022 results**

#5

#1 AND #2 AND #3 AND #4

[1,022](https://www-embase-com.ezproxy.library.qmul.ac.uk/)

#4

“low- and middle-income countries” OR lmics OR ”low and middle income countries”/exp OR ”low and middle income countries” OR ”afghanistan”/exp OR afghanistan OR ”gambia”/exp OR ”gambia” OR ”niger”/exp OR niger OR ”benin”/exp OR benin OR ”guinea”/exp OR guinea OR ”rwanda”/exp OR rwanda OR ”burkina faso”/exp OR ”burkina faso”OR ”sierra leone”/exp OR ”sierra leone” OR ”burundi”/exp OR burundi OR ”haiti”/exp OR haiti OR ”somalia”/exp OR somalia OR ”cambodia”/exp OR cambodia OR ”korea”/exp OR korea OR ”south sudan”/exp OR ”south sudan” OR ”central african republic”/exp OR ”central african republic” OR ”liberia”/exp OR liberia OR ”tanzania”/exp OR tanzania OR ”chad”/exp OR chad OR ”madagascar”/exp OR madagascar OR ”togo”/exp OR togo OR ”comoros”/exp OR comoros OR ”malawi”/exp OR malawi OR ”uganda”/exp OR uganda OR ”mali”/exp OR mali OR ”zimbabwe”/exp OR zimbabwe OR ”eritrea”/exp OR eritrea OR ”mozambique”/exp OR mozambique OR ”ethiopia”/exp OR ethiopia OR ”nepal”/exp OR nepalOR ”armenia”/exp OR armenia OR ”indonesia”/exp OR indonesia OR ”samoa”/exp OR samoa OR ”bangladesh”/exp OR bangladesh OR ”kenya”/exp OR kenya OR ”bhutan”/exp OR bhutan OR ”kiribati”/exp OR kiribati OR ”senegal”/exp OR senegal OR ”bolivia”/exp OR bolivia OR ”kosovo”/exp OR kosovo OR ”solomon islands”/exp OR ”solomon islands” OR ”cabo verde”/exp OR ”cabo verde” OR ”kyrgyzstan”/exp OR kyrgyzstan OR ”sri lanka”/exp OR ”sri lanka” OR ”cameroon”/exp OR cameroon OR ”sudan”/exp OR sudan OR ”congo”/exp OR congo OR ”lesotho”/exp OR lesotho OR ”swaziland”/exp OR swaziland OR ”cote divoire” OR ”mauritania”/exp OR mauritania OR ”syrian arab republic”/exp OR ”syrian arab republic” OR ”djibouti”/exp OR djibouti OR ”micronesia”/exp OR micronesia OR ”tajikistan”/exp OR tajikistan OR ”egypt”/exp OR egypt OR ”moldova”/exp OR moldova OR ”timor leste”/exp OR ”timor leste” OR ”el salvador”/exp OR ”el salvador” OR ”morocco”/exp OR morocco OR ”ukraine”/exp OR ukraine OR ”georgia”/exp OR georgiaOR ”myanmar”/exp OR myanmar OR ”uzbekistan”/exp OR uzbekistan OR ”ghana”/exp OR ghana OR ”nicaragua”/exp OR nicaragua OR ”vanuatu”/exp OR vanuatu OR ”guatemala”/exp OR guatemala OR ”nigeria”/exp OR nigeria OR ”vietnam”/exp OR vietnam OR ”guyana”/exp OR guyana OR ”pakistan”/exp OR pakistan OR ”west bank and gaza”OR ”honduras”/exp OR honduras OR ”papua new guinea”/exp OR ”papua new guinea” OR ”yemen”/exp OR yemen OR ”india”/exp OR india OR ”philippines”/exp OR philippinesOR ”zambia”/exp OR zambia OR ”albania”/exp OR albania OR ”fiji”/exp OR fiji OR ”namibia”/exp OR namibia OR ”algeria”/exp OR algeria OR ”gabon”/exp OR gabon OR ”palau”/exp OR palau OR ”american samoa”/exp OR ”american samoa” OR ”grenada”/exp OR grenada OR ”panama”/exp OR panama OR ”angola”/exp OR angola OR ”iran”/exp OR iran OR ”paraguay”/exp OR paraguay OR ”azerbaijan”/exp OR azerbaijan OR ”iraq”/exp OR iraq OR ”peru”/exp OR peru OR ”belarus”/exp OR belarus OR ”jamaica”/exp OR jamaica OR ”romania”/exp OR romania OR ”belize”/exp OR belize OR ”jordan”/exp OR jordan OR ”serbia”/exp OR serbia OR ”bosnia and herzegovina”/exp OR ”bosnia and herzegovina” OR ”kazakhstan”/exp OR kazakhstan OR ”south africa”/exp OR ”south africa” OR ”botswana”/exp OR botswana OR ”lebanon”/exp OR lebanon OR ”st. lucia”/exp OR ”st. lucia” OR ”brazil”/exp OR brazil OR ”libya”/exp OR libya OR ”st. vincent and the grenadines”/exp OR ”st. vincent and the grenadines” OR ”bulgaria”/exp OR bulgaria OR macedonia OR ”suriname”/exp OR suriname OR ”china”/exp OR china OR ”malaysia”/exp OR malaysia OR ”thailand”/exp OR thailand OR ”colombia”/exp OR colombia OR ”maldives”/exp OR maldives OR ”tonga”/exp OR tonga OR ”costa rica”/exp OR ”costa rica” OR ”marshall islands”/exp OR ”marshall islands” OR ”tunisia”/exp OR tunisia OR ”cuba”/exp OR cuba OR ”mauritius”/exp OR mauritius OR ”turkey”/exp OR turkey OR ”dominica”/exp OR dominica OR ”mexico”/exp OR mexico OR ”turkmenistan”/exp OR turkmenistan OR ”dominican republic”/exp OR ”dominican republic” OR ”mongolia”/exp OR mongolia OR ”tuvalu”/exp OR tuvalu OR ”ecuador”/exp OR ecuador OR montenegro:ab

[8,472,177](https://www-embase-com.ezproxy.library.qmul.ac.uk/)

#3

“maternal healthcare services” OR ”reproductive healthcare services” OR ”child healthcare services” OR ”reproductive health”/exp OR ”reproductive health” OR ”maternal health”/exp OR ”maternal health” OR ”child health”/exp OR ”child health” OR ”newborn health”/exp OR ”newborn health” OR ”family planning”/exp OR ”family planning” OR ”pregnancy”/exp OR pregnancy OR ”birth control”/exp OR ”birth control” OR abortion:ab

[1,508,535](https://www-embase-com.ezproxy.library.qmul.ac.uk/)

#2

“covid 19”/exp OR ”covid 19” OR ”covid”/exp OR covid OR ”covid19”/exp OR covid19 OR ”coronavirus”/exp OR coronavirus OR ”sars-cov-2”/exp OR ”sars-cov-2” OR ”2019-ncov”:ab

[220,482](https://www-embase-com.ezproxy.library.qmul.ac.uk/)

#1

“impact”/exp OR impact OR effect OR influence OR ”consequence*” OR significance:ab

[9,504,988](https://www-embase-com.ezproxy.library.qmul.ac.uk/)

1. Web of Science - conducted on 15^th^ of October 2021 – **565 results**

5

**(((#1) AND #2) AND #3) AND #4**

[565](https://www-webofscience-com.ezproxy.library.qmul.ac.uk/wos/woscc/summary/4ba85e5b-4b61-4f4e-ad0b-332e29337a85-0d37d476/relevance/1)

4

**AB=(“low- and middle-income countries” OR LMICs OR “low and middle income countries” OR Afghanistan OR “Gambia” OR Niger OR Benin OR Guinea OR Rwanda OR “Burkina Faso” OR “Sierra Leone” OR Burundi OR Haiti OR Somalia OR Cambodia OR Korea OR “South Sudan” OR “Central African Republic” OR Liberia OR Tanzania OR Chad OR Madagascar OR Togo OR Comoros OR Malawi OR Uganda OR Congo OR Mali OR Zimbabwe OR Eritrea OR Mozambique OR Ethiopia OR Nepal OR Armenia OR Indonesia OR Samoa OR Bangladesh OR Kenya OR Bhutan OR Kiribati OR Senegal OR Bolivia OR Kosovo OR “Solomon Islands” OR “Cabo Verde” OR Kyrgyzstan OR “Sri Lanka” OR Cameroon OR Sudan OR Congo OR Lesotho OR Swaziland OR “Cote divoire” OR Mauritania OR “Syrian Arab Republic” OR Djibouti OR Micronesia OR Tajikistan OR Egypt OR Moldova OR Timor-Leste OR “El Salvador” OR Morocco OR Ukraine OR Georgia OR Myanmar OR Uzbekistan OR Ghana OR Nicaragua OR Vanuatu OR Guatemala OR Nigeria OR Vietnam OR Guyana OR Pakistan OR “West Bank and Gaza” OR Honduras OR “Papua New Guinea” OR Yemen OR India OR Philippines OR Zambia OR Albania OR Fiji OR Namibia OR Algeria OR Gabon OR Palau OR “American Samoa” OR Grenada OR Panama OR Angola OR Iran OR Paraguay OR Azerbaijan OR Iraq OR Peru OR Belarus OR Jamaica OR Romania OR Belize OR Jordan OR Serbia OR “Bosnia and Herzegovina” OR Kazakhstan OR “South Africa” OR Botswana OR Lebanon OR “St. Lucia” OR Brazil OR Libya OR “St. Vincent and the Grenadines” OR Bulgaria OR Macedonia OR Suriname OR China OR Malaysia OR Thailand OR Colombia OR Maldives OR Tonga OR “Costa Rica” OR “Marshall Islands” OR Tunisia OR Cuba OR Mauritius OR Turkey OR Dominica OR Mexico OR Turkmenistan OR “Dominican Republic” OR Mongolia OR Tuvalu OR Ecuador OR Montenegro)**

[2,196,001](https://www-webofscience-com.ezproxy.library.qmul.ac.uk/wos/woscc/summary/ee98c6e3-72ba-42d2-99cb-0015d60d6029-0d37b244/relevance/1)

3

**AB=(“maternal healthcare services” OR “reproductive healthcare services” OR “child healthcare services” OR “reproductive health” OR “maternal health” OR “child health” OR “newborn health” OR “family planning” OR pregnancy OR “birth control” OR abortion)**

[585,685](https://www-webofscience-com.ezproxy.library.qmul.ac.uk/wos/woscc/summary/6a34a30b-9197-44f5-936f-8a248a6c78e7-0d37af81/relevance/1)

2

**AB=(COVID-19 OR COVID OR COVID19 OR coronavirus OR “SARS-CoV-2” OR “2019-nCoV”)**

[143,987](https://www-webofscience-com.ezproxy.library.qmul.ac.uk/wos/woscc/summary/620c2d2a-a6fa-424a-b966-f460b5ea7881-0d37a464/relevance/1)

1

**AB=(impact OR effect OR influence OR “consequence*” OR significance)**

[12,681,480](https://www-webofscience-com.ezproxy.library.qmul.ac.uk/wos/woscc/summary/ed6cc9f4-c2c1-4c72-b036-2ac03df4a078-0d37a114/relevance/1)

1. The Cochrane Central Register of Controlled Trials - conducted on 15^th^ of October 2021 - **227 results**

#1

impact OR effect OR influence OR ‘consequence*’ OR significance

#2

COVID-19 OR COVID OR COVID19 OR coronavirus OR "SARS-CoV-2" OR "2019-nCoV"

#3

“maternal healthcare services” OR “reproductive healthcare services” OR “child healthcare services” OR “reproductive health” OR “maternal health” OR “child health” OR “newborn health” OR “family planning” OR pregnancy OR “birth control” OR abortion

227

227

1. Bottom of Form

SEARCH UPDATE

29/06/2023

Pubmed – 399

**#6**

**Search: #1 AND #2 AND #3 AND #4 Filters: from 2021/10/16 - 2023/6/29**

[**399**](https://pubmed.ncbi.nlm.nih.gov/?term=%231+AND+%232+AND+%233+AND+%234&filter=dates.2021%2F10%2F16-2023%2F6%2F29&sort=relevance)**00:31:55#5**

**Search: #1 AND #2 AND #3 AND #4**

[**621**](https://pubmed.ncbi.nlm.nih.gov/?term=%231+AND+%232+AND+%233+AND+%234&sort=)**00:31:16#4**

**Search: "low- and middle-income countries"[Title/Abstract] OR LMICs[Title/Abstract] OR "low and middle income countries"[Title/Abstract] OR Afghanistan[Title/Abstract] OR "Gambia"[Title/Abstract] OR Niger[Title/Abstract] OR Benin[Title/Abstract] OR Guinea[Title/Abstract] OR Rwanda[Title/Abstract] OR "Burkina Faso"[Title/Abstract] OR "Sierra Leone"[Title/Abstract] OR Burundi[Title/Abstract] OR Haiti[Title/Abstract] OR Somalia[Title/Abstract] OR Cambodia[Title/Abstract] OR Korea[Title/Abstract] OR "South Sudan"[Title/Abstract] OR "Central African Republic"[Title/Abstract] OR Liberia[Title/Abstract] OR Tanzania[Title/Abstract] OR Chad[Title/Abstract] OR Madagascar[Title/Abstract] OR Togo[Title/Abstract] OR Comoros[Title/Abstract] OR Malawi[Title/Abstract] OR Uganda[Title/Abstract] OR Congo[Title/Abstract] OR Mali[Title/Abstract] OR Zimbabwe[Title/Abstract] OR Eritrea[Title/Abstract] OR Mozambique[Title/Abstract] OR Ethiopia[Title/Abstract] OR Nepal[Title/Abstract] OR Armenia[Title/Abstract] OR Indonesia[Title/Abstract] OR Samoa[Title/Abstract] OR Bangladesh[Title/Abstract] OR Kenya[Title/Abstract] OR Bhutan[Title/Abstract] OR Kiribati[Title/Abstract] OR Senegal[Title/Abstract] OR Bolivia[Title/Abstract] OR Kosovo[Title/Abstract] OR "Solomon Islands"[Title/Abstract] OR "Cabo Verde"[Title/Abstract] OR Kyrgyzstan[Title/Abstract] OR "Sri Lanka"[Title/Abstract] OR Cameroon[Title/Abstract] OR Sudan[Title/Abstract] OR Congo[Title/Abstract] OR Lesotho[Title/Abstract] OR Swaziland[Title/Abstract] OR "Cote divoire"[Title/Abstract] OR Mauritania[Title/Abstract] OR "Syrian Arab Republic"[Title/Abstract] OR Djibouti[Title/Abstract] OR Micronesia[Title/Abstract] OR Tajikistan[Title/Abstract] OR Egypt[Title/Abstract] OR Moldova[Title/Abstract] OR Timor-Leste[Title/Abstract] OR "El Salvador"[Title/Abstract] OR Morocco[Title/Abstract] OR Ukraine[Title/Abstract] OR Georgia[Title/Abstract] OR Myanmar[Title/Abstract] OR Uzbekistan[Title/Abstract] OR Ghana[Title/Abstract] OR Nicaragua[Title/Abstract] OR Vanuatu[Title/Abstract] OR Guatemala[Title/Abstract] OR Nigeria[Title/Abstract] OR Vietnam[Title/Abstract] OR Guyana[Title/Abstract] OR Pakistan[Title/Abstract] OR "West Bank and Gaza"[Title/Abstract] OR Honduras[Title/Abstract] OR "Papua New Guinea"[Title/Abstract] OR Yemen[Title/Abstract] OR India[Title/Abstract] OR Philippines[Title/Abstract] OR Zambia[Title/Abstract] OR Albania[Title/Abstract] OR Fiji[Title/Abstract] OR Namibia[Title/Abstract] OR Algeria[Title/Abstract] OR Gabon[Title/Abstract] OR Palau[Title/Abstract] OR "American Samoa"[Title/Abstract] OR Grenada[Title/Abstract] OR Panama[Title/Abstract] OR Angola[Title/Abstract] OR Iran[Title/Abstract] OR Paraguay[Title/Abstract] OR Azerbaijan[Title/Abstract] OR Iraq[Title/Abstract] OR Peru[Title/Abstract] OR Belarus[Title/Abstract] OR Jamaica[Title/Abstract] OR Romania[Title/Abstract] OR Belize[Title/Abstract] OR Jordan[Title/Abstract] OR Serbia[Title/Abstract] OR "Bosnia and Herzegovina"[Title/Abstract] OR Kazakhstan[Title/Abstract] OR "South Africa"[Title/Abstract] OR Botswana[Title/Abstract] OR Lebanon[Title/Abstract] OR Brazil[Title/Abstract] OR Libya[Title/Abstract] OR Bulgaria[Title/Abstract] OR Macedonia[Title/Abstract] OR Suriname[Title/Abstract] OR China[Title/Abstract] OR Malaysia[Title/Abstract] OR Thailand[Title/Abstract] OR Colombia[Title/Abstract] OR Maldives[Title/Abstract] OR Tonga[Title/Abstract] OR "Costa Rica"[Title/Abstract] OR "Marshall Islands"[Title/Abstract] OR Tunisia[Title/Abstract] OR Cuba[Title/Abstract] OR Mauritius[Title/Abstract] OR Turkey[Title/Abstract] OR Dominica[Title/Abstract] OR Mexico[Title/Abstract] OR Turkmenistan[Title/Abstract] OR "Dominican Republic"[Title/Abstract] OR Mongolia[Title/Abstract] OR Tuvalu[Title/Abstract] OR Ecuador[Title/Abstract] OR Montenegro[Title/Abstract]**

[**1,427,909**](https://pubmed.ncbi.nlm.nih.gov/?term=longqueryedaabc71cace2f44a6e5&sort=)**00:28:58#3**

**Search: "maternal healthcare services"[Title/Abstract] OR "reproductive healthcare services"[Title/Abstract] OR "child healthcare services"[Title/Abstract] OR "reproductive health"[Title/Abstract] OR "maternal health"[Title/Abstract] OR "child health"[Title/Abstract] OR "newborn health"[Title/Abstract] OR "family planning"[Title/Abstract] OR pregnancy[Title/Abstract] OR "birth control"[Title/Abstract] OR abortion[Title/Abstract]**

[**582,904**](https://pubmed.ncbi.nlm.nih.gov/?term=%22maternal+healthcare+services%22%5BTitle%2FAbstract%5D+OR+%22reproductive+healthcare+services%22%5BTitle%2FAbstract%5D+OR+%22child+healthcare+services%22%5BTitle%2FAbstract%5D+OR+%22reproductive+health%22%5BTitle%2FAbstract%5D+OR+%22maternal+health%22%5BTitle%2FAbstract%5D+OR+%22child+health%22%5BTitle%2FAbstract%5D+OR+%22newborn+health%22%5BTitle%2FAbstract%5D+OR+%22family+planning%22%5BTitle%2FAbstract%5D+OR+pregnancy%5BTitle%2FAbstract%5D+OR+%22birth+control%22%5BTitle%2FAbstract%5D+OR+abortion%5BTitle%2FAbstract%5D&sort=)**00:28:45#2**

**Search: COVID-19[Title/Abstract] OR COVID[Title/Abstract] OR COVID19[Title/Abstract] OR coronavirus[Title/Abstract] OR "SARS-CoV-2"[Title/Abstract] OR "2019-nCoV"[Title/Abstract]**

[**371,977**](https://pubmed.ncbi.nlm.nih.gov/?term=COVID-19%5BTitle%2FAbstract%5D+OR+COVID%5BTitle%2FAbstract%5D+OR+COVID19%5BTitle%2FAbstract%5D+OR+coronavirus%5BTitle%2FAbstract%5D+OR+%22SARS-CoV-2%22%5BTitle%2FAbstract%5D+OR+%222019-nCoV%22%5BTitle%2FAbstract%5D&sort=)**00:28:33#1**

**Search: impact[Title/Abstract] OR effect[Title/Abstract] OR influence[Title/Abstract] OR "consequence*"[Title/Abstract] OR significance[Title/Abstract]**

[**6,684,118**](https://pubmed.ncbi.nlm.nih.gov/?term=impact%5BTitle%2FAbstract%5D+OR+effect%5BTitle%2FAbstract%5D+OR+influence%5BTitle%2FAbstract%5D+OR+%22consequence%2A%22%5BTitle%2FAbstract%5D+OR+significance%5BTitle%2FAbstract%5D&sort=)**00:28:15**

Scopus – 733

- 8

[Edit](https://www.scopus.com/search/history/edit.uri?shid=8)

( TITLE-ABS-KEY ( impact OR effect OR influence OR "consequence*" OR significance ) ) AND ( TITLE-ABS-KEY ( covid-19 OR covid OR covid19 OR coronavirus OR "sars-cov-2" OR "2019-ncov" ) ) AND ( TITLE-ABS-KEY ( "maternal healthcare services" OR "reproductive healthcare services" OR "child healthcare services" OR "reproductive health" OR "maternal health" OR "child health" OR "newborn health" OR "family planning" OR pregnancy OR "birth control" OR abortion ) ) AND ( TITLE-ABS-KEY ( "low- and middle-income countries" OR lmics OR "low and middle income countries" OR afghanistan OR "gambia" OR niger OR benin OR guinea OR rwanda OR "burkina faso" OR "sierra leone" OR burundi OR haiti OR somalia OR cambodia OR korea OR "south sudan" OR "central african republic" OR liberia OR tanzania OR chad OR madagascar OR togo OR comoros OR malawi OR uganda OR congo OR mali OR zimbabwe OR eritrea OR mozambique OR ethiopia OR nepal OR armenia OR indonesia OR samoa OR bangladesh OR kenya OR bhutan OR kiribati OR senegal OR bolivia OR kosovo OR "solomon islands" OR "cabo verde" OR kyrgyzstan OR "sri lanka" OR cameroon OR sudan OR congo OR lesotho OR swaziland OR "cote divoire" OR mauritania OR "syrian arab republic" OR djibouti OR micronesia OR tajikistan OR egypt OR moldova OR timor-leste OR "el salvador" OR morocco OR ukraine OR georgia OR myanmar OR uzbekistan OR ghana OR nicaragua OR vanuatu OR guatemala OR nigeria OR vietnam OR guyana OR pakistan OR "west bank and gaza" OR honduras OR "papua new guinea" OR yemen OR india OR philippines OR zambia OR albania OR fiji OR namibia OR algeria OR gabon OR palau OR "american samoa" OR grenada OR panama OR angola OR iran OR paraguay OR azerbaijan OR iraq OR peru OR belarus OR jamaica OR romania OR belize OR jordan OR serbia OR "bosnia and herzegovina" OR kazakhstan OR "south africa" OR botswana OR lebanon OR brazil OR libya OR bulgaria OR macedonia OR suriname OR china OR malaysia OR thailand OR colombia OR maldives OR tonga OR "costa rica" OR "marshall islands" OR tunisia OR cuba OR mauritius OR turkey OR dominica OR mexico OR turkmenistan OR "dominican republic" OR mongolia OR tuvalu OR ecuador OR montenegro ) ) AND ( LIMIT-TO ( PUBYEAR , 2022 ) OR LIMIT-TO ( PUBYEAR , 2023 ) )

Show less

[733 results](https://www.scopus.com/search/history/results.uri?origin=searchhistory&shid=8)

Set AlertMore

- 6

[Edit](https://www.scopus.com/search/history/edit.uri?shid=6)

( TITLE-ABS-KEY ( impact OR effect OR influence OR "consequence*" OR significance ) ) AND ( TITLE-ABS-KEY ( covid-19 OR covid OR covid19 OR coronavirus OR "sars-cov-2" OR "2019-ncov" ) ) AND ( TITLE-ABS-KEY ( "maternal healthcare services" OR "reproductive healthcare services" OR "child healthcare services" OR "reproductive health" OR "maternal health" OR "child health" OR "newborn health" OR "family planning" OR pregnancy OR "birth control" OR abortion ) ) AND ( TITLE-ABS-KEY ( "low- and middle-income countries" OR lmics OR "low and middle income countries" OR afghanistan OR "gambia" OR niger OR benin OR guinea OR rwanda OR "burkina faso" OR "sierra leone" OR burundi OR haiti OR somalia OR cambodia OR korea OR "south sudan" OR "central african republic" OR liberia OR tanzania OR chad OR madagascar OR togo OR comoros OR malawi OR uganda OR congo OR mali OR zimbabwe OR eritrea OR mozambique OR ethiopia OR nepal OR armenia OR indonesia OR samoa OR bangladesh OR kenya OR bhutan OR kiribati OR senegal OR bolivia OR kosovo OR "solomon islands" OR "cabo verde" OR kyrgyzstan OR "sri lanka" OR cameroon OR sudan OR congo OR lesotho OR swaziland OR "cote divoire" OR mauritania OR "syrian arab republic" OR djibouti OR micronesia OR tajikistan OR egypt OR moldova OR timor-leste OR "el salvador" OR morocco OR ukraine OR georgia OR myanmar OR uzbekistan OR ghana OR nicaragua OR vanuatu OR guatemala OR nigeria OR vietnam OR guyana OR pakistan OR "west bank and gaza" OR honduras OR "papua new guinea" OR yemen OR india OR philippines OR zambia OR albania OR fiji OR namibia OR algeria OR gabon OR palau OR "american samoa" OR grenada OR panama OR angola OR iran OR paraguay OR azerbaijan OR iraq OR peru OR belarus OR jamaica OR romania OR belize OR jordan OR serbia OR "bosnia and herzegovina" OR kazakhstan OR "south africa" OR botswana OR lebanon OR brazil OR libya OR bulgaria OR macedonia OR suriname OR china OR malaysia OR thailand OR colombia OR maldives OR tonga OR "costa rica" OR "marshall islands" OR tunisia OR cuba OR mauritius OR turkey OR dominica OR mexico OR turkmenistan OR "dominican republic" OR mongolia OR tuvalu OR ecuador OR montenegro ) )

Show less

[1,361 results](https://www.scopus.com/search/history/results.uri?origin=searchhistory&shid=6)

Set AlertMore

- 4

[Edit](https://www.scopus.com/search/history/edit.uri?shid=4)

TITLE-ABS-KEY ( "low- and middle-income countries" OR lmics OR "low and middle income countries" OR afghanistan OR "gambia" OR niger OR benin OR guinea OR rwanda OR "burkina faso" OR "sierra leone" OR burundi OR haiti OR somalia OR cambodia OR korea OR "south sudan" OR "central african republic" OR liberia OR tanzania OR chad OR madagascar OR togo OR comoros OR malawi OR uganda OR congo OR mali OR zimbabwe OR eritrea OR mozambique OR ethiopia OR nepal OR armenia OR indonesia OR samoa OR bangladesh OR kenya OR bhutan OR kiribati OR senegal OR bolivia OR kosovo OR "solomon islands" OR "cabo verde" OR kyrgyzstan OR "sri lanka" OR cameroon OR sudan OR congo OR lesotho OR swaziland OR "cote divoire" OR mauritania OR "syrian arab republic" OR djibouti OR micronesia OR tajikistan OR egypt OR moldova OR timor-leste OR "el salvador" OR morocco OR ukraine OR georgia OR myanmar OR uzbekistan OR ghana OR nicaragua OR vanuatu OR guatemala OR nigeria OR vietnam OR guyana OR pakistan OR "west bank and gaza" OR honduras OR "papua new guinea" OR yemen OR india OR philippines OR zambia OR albania OR fiji OR namibia OR algeria OR gabon OR palau OR "american samoa" OR grenada OR panama OR angola OR iran OR paraguay OR azerbaijan OR iraq OR peru OR belarus OR jamaica OR romania OR belize OR jordan OR serbia OR "bosnia and herzegovina" OR kazakhstan OR "south africa" OR botswana OR lebanon OR brazil OR libya OR bulgaria OR macedonia OR suriname OR china OR malaysia OR thailand OR colombia OR maldives OR tonga OR "costa rica" OR "marshall islands" OR tunisia OR cuba OR mauritius OR turkey OR dominica OR mexico OR turkmenistan OR "dominican republic" OR mongolia OR tuvalu OR ecuador OR montenegro )

Show less

[5,394,891 results](https://www.scopus.com/search/history/results.uri?origin=searchhistory&shid=4)

Set AlertMore

- 3

[Edit](https://www.scopus.com/search/history/edit.uri?shid=3)

TITLE-ABS-KEY ( "maternal healthcare services" OR "reproductive healthcare services" OR "child healthcare services" OR "reproductive health" OR "maternal health" OR "child health" OR "newborn health" OR "family planning" OR pregnancy OR "birth control" OR abortion )

Show less

[1,423,181 results](https://www.scopus.com/search/history/results.uri?origin=searchhistory&shid=3)

Set AlertMore

- 2

[Edit](https://www.scopus.com/search/history/edit.uri?shid=2)

TITLE-ABS-KEY ( covid-19 OR covid OR covid19 OR coronavirus OR "sars-cov-2" OR "2019-ncov" )

[561,831 results](https://www.scopus.com/search/history/results.uri?origin=searchhistory&shid=2)

Set AlertMore

- 1

[Edit](https://www.scopus.com/search/history/edit.uri?shid=1)

TITLE-ABS-KEY ( impact OR effect OR influence OR "consequence*" OR significance )

[23,961,258 results](https://www.scopus.com/search/history/results.uri?origin=searchhistory&shid=1)

Set AlertMore
