# Supplementary material for: Impact of the COVID-19 pandemic on access to and delivery of maternal and child healthcare services in low-and middle-income countries: a systematic review of the literature
Source: Front Public Health. 2024 Apr 8;12:1346268. doi: 10.3389/fpubh.2024.1346268 (PMC11036866; doi:10.3389/fpubh.2024.1346268)
Supplement: Supplementary file 3 [file Data_Sheet_3.pdf]

Quality assessment of cross-sectional studies	Author(s), year	1) Was the research question or objective in this paper clearly stated?	2) Was the study population clearly specified and defined?	3) Was the participation rate of eligible persons at least 50%?	4) Were all the subjects selected or recruited from the same or similar populations (including the same time period)? Were inclusion and exclusion criteria for being in the study prespecified and applied uniformly to all participants?	5) Was a sample size justification, power description, or variance and effect estimates provided?	9) Were the exposure measures (independent variables) clearly defined, valid, reliable, and implemented consistently across all study participants?	10) Was the exposure(s) assessed more than once over time?	11) Were the outcome measures (dependent variables) clearly defined, valid, reliable, and implemented consistently across all study participants?	14) Were key potential confounding variables measured and adjusted statistically for their impact on the relationship between exposure(s) and outcome (s)?					6) For the analyses in this paper, were the exposure(s) of interest measured prior to the outcome(s) being measured?	7) Was the timeframe sufficient so that one could reasonably expect to see an association between exposure and outcome if it existed?	8) For exposures that can vary in amount or level, did the study examine different levels of the exposure as related to the outcome (e.g., categories of exposure, or exposure measured as continuous variable)?	12) Were the outcome assessors blinded to the exposure status of participants?	13) Was loss to follow-up after baseline 20% or less?
1	Abdul-Mumin et al., 2021	yes	yes	not applicable	yes	not applicable	yes	no	yes	not reported									
2	Akuaake et al., 2020	yes	yes	not applicable	yes	not applicable	yes	no	yes	not reported									
3	Assefa et al., 2021	yes	yes	not reported	yes	not reported	yes	no	yes	yes									
4	Shakespeare et al., 2021	yes	yes	not applicable	yes	yes	yes	no	yes	yes									
5	Muhaidat et al., 2020	yes	yes	yes	yes	yes	yes	no	yes	not reported									
6	Ogundele et al., 2020	yes	yes	yes	yes	not reported	yes	no	yes	not reported									
7	Enyama et al., 2020	yes	yes	yes	yes	not reported	yes	no	yes	not reported									
8	Enbiale et al., 2021	yes	yes	not reported	yes	not reported	yes	no	yes	not reported									
9	Mhajabin et al., 2022	yes	yes	yes	yes	yes	yes	no	yes	not reported									
10	Goyal et al., 2022	yes	yes	not reported	yes	yes	yes	no	yes	not reported									
11	Gebreegziabher et al., 2022	yes	yes	not reported	yes	not reported	yes	no	yes	not reported									

Quality assessment of pre-post studies	Author(s), year	1. Was the study question or objective clearly stated?	2. Were eligibility/selection criteria for the study population prespecified and clearly described?	3. Were the participants in the study representative of those who would be eligible for the test/service/intervention in the general or clinical population of interest?	4. Were all eligible participants that met the prespecified entry criteria enrolled?	5. Was the sample size sufficiently large to provide confidence in the findings?	6. Was the test/service/intervention clearly described and delivered consistently across the study population?	7. Were the outcome measures prespecified, clearly defined, valid, reliable, and assessed consistently across all study participants?	8. Were the people assessing the outcomes blinded to the participants' exposures/interventions?	9. Was the loss to follow-up after baseline 20% or less? Were those lost to follow-up accounted for in the analysis?	10. Did the statistical methods examine changes in outcome measures from before to after the intervention? Were statistical tests done that provided p values for the pre-to-post changes?	11. Were outcome measures of interest taken multiple times before the intervention and multiple times after the intervention (i. e., did they use an interrupted time-series design)?	12. If the intervention was conducted at a group level (e.g., a whole hospital, a community, etc.) did the statistical analysis take into account the use of individual-level data to determine effects at the group level?
1	Abdela et al., 2020	yes	yes	yes	yes	cannot determine	yes	yes	not applicable	not applicable	not reported	yes	no
2	Ahmed et al., 2021	yes	yes	yes	yes	cannot determine	yes	yes	not applicable	not applicable	not reported	no	no
3	Baloch et al., 2021	yes	yes	cannot determine	yes	cannot determine	yes	yes	not applicable	not applicable	not reported	yes	no
4	Singh et al., 2021	yes	yes	cannot determine	yes	cannot determine	yes	yes	not applicable	not applicable	not reported	yes	no
5	Rahul et al., 2020	yes	yes	yes	yes	cannot determine	yes	yes	not applicable	not applicable	not reported	no	no
6	Qureshi et al., 2021	yes	yes	cannot determine	yes	cannot determine	yes	yes	not applicable	not applicable	yes	no	no
7	Caniglia et al., 2021	yes	yes	cannot determine	yes	cannot determine	yes	yes	not applicable	not applicable	not reported	no	no
8	Desta et al., 2021	yes	yes	cannot determine	yes	cannot determine	yes	yes	not applicable	not applicable	yes	no	no
9	Pillay et al., 2021	yes	yes	cannot determine	yes	cannot determine	yes	yes	not applicable	not applicable	not reported	no	no
10	Goyal et al., 2021	yes	yes	cannot determine	yes	cannot determine	yes	yes	not applicable	not applicable	yes	no	no
11	Sharma et al., 2023	yes	yes	yes	yes	cannot determine	yes	yes	not applicable	not applicable	not reported	yes	no
12	Requena-Mullor et al., 2022	yes	yes	yes	yes	yes	yes	yes	not applicable	not applicable	yes	no	no
13	Millimouno et al., 2023	yes	yes	cannot determine	yes	cannot determine	yes	yes	not applicable	not applicable	yes	no	no
14	Thsehla et al., 2023	yes	yes	cannot determine	yes	cannot determine	yes	yes	not applicable	not applicable	yes	no	no

Quality assessment of time series studies	Author(s), year	1. Was the study question or objective clearly stated?	2. Was the study population clearly and fully described, including a case definition?	3. Were the cases consecutive?	4. Were the subjects comparable?	5. Was the intervention clearly described?	6. Were the outcome measures clearly defined, valid, reliable, and implemented consistently across all study participants?	7. Was the length of follow-up adequate?	8. Were the statistical methods well-described?	9. Were the results well-described?
1	Abebe et al., 2021	yes	yes	yes	yes	yes	yes	cannot determine	yes	yes
2	Shapira et al., 2021	yes	yes	yes	yes	yes	yes	cannot determine	yes	yes
3	Doubova et al., 2021	yes	yes	yes	yes	yes	yes	cannot determine	yes	yes
4	Burt et al., 2021	yes	yes	yes	yes	yes	yes	cannot determine	yes	yes
5	Hategeka et al., 2021	yes	yes	yes	yes	yes	yes	cannot determine	yes	yes
6	Yadollahi et al., 2022	yes	yes	yes	yes	yes	yes	cannot determine	yes	yes
7	Tikouk et al., 2023	yes	yes	yes	yes	yes	yes	cannot determine	yes	yes
8	Lydon et el., 2022	yes	yes	yes	yes	yes	yes	cannot determine	yes	yes
9	Emmanuel et al., 2022	yes	yes	yes	yes	yes	yes	cannot determine	yes	yes

Quality assessment of mixed-methods studies	Author(s), year	1. Are there clear research questions?	2. Do the collected data allow to address the research questions?	3. Is there an adequate rationale for using a mixed methods design to address the research question?	4. Are the different components of the study effectively integrated to answer the research question?	5. Are the outputs of the integration of qualitative and quantitative components adequately interpreted?	6. Are divergences and inconsistencies between quantitative and qualitative results adequately addressed?	7. Do the different components of the study adhere to the quality criteria of each tradition of the methods involved?
1	Pires et al., 2021	yes	yes	cannot determine	yes	yes	cannot determine	yes
2	Tilahun et al., 2022	yes	yes	cannot determine	yes	yes	cannot determine	yes
3	Sinha et al., 2022	yes	yes	yes	yes	yes	cannot determine	no
4	Padhye et al., 2022	yes	yes	cannot determine	yes	no	cannot determine	no
5	Bekele et al., 2022	yes	yes	yes	yes	yes	yes	yes

Quality assessment of qualitative studies	Author(s), year	1. Was there a clear statement of the aims of the research?	2. Is a qualitative methodology appropriate?	3. Was the research design appropriate to address the aims of the research?	4. Was the recruitment strategy appropriate to the aims of the research?	5. Was the data collected in a way that addressed the research issue?	6. Has the relationship between researcher and participants been adequately considered?	7. Have ethical issues been taken into consideration?	8. Was the data analysis sufficiently rigorous?	9. Is there a clear statement of findings?	10. How valuable is the research?
1	Onchonga et al., 2021	yes	yes	yes	yes	yes	cannot determine	yes	yes	yes	cannot determine
2	Hailemariam et al., 2021	yes	yes	yes	yes	yes	yes	yes	yes	yes	cannot determine
3	Thahir et al., 2023	yes	yes	yes	cannot determine	yes	yes	yes	yes	yes	cannot determine
4	Kabagenyi et al., 2022	yes	yes	yes	cannot determine	yes	cannot determine	yes	yes	yes	cannot determine
5	Bliznashka et al., 2022	yes	yes	yes	cannot determine	yes	cannot determine	yes	yes	yes	cannot determine
6	Basnet et al., 2022	yes	yes	yes	cannot determine	yes	cannot determine	yes	yes	yes	cannot determine
